# Bu-Shen-Huo-Xue Decoction Ameliorates Diabetic Nephropathy by Inhibiting Rac1/PAK1/p38MAPK Signaling Pathway in High-Fat Diet/Streptozotocin-Induced Diabetic Mice

**DOI:** 10.3389/fphar.2020.587663

**Published:** 2020-12-03

**Authors:** Weisong Wang, Hongping Long, Wei Huang, Ting Zhang, Lihua Xie, Cheng Chen, Jianhe Liu, Dan Xiong, Wei Hu

**Affiliations:** ^1^ Graduate School, Hunan University of Chinese Medicine, Changsha, China; ^2^ Experiment Center of Medical Innovation, The First Hospital of Hunan University of Chinese Medicine, Changsha, China; ^3^ Department of Cardiology, The First Hospital of Hunan University of Chinese Medicine, Changsha, China; ^4^ Department of Nephrology, The First Hospital of Hunan University of Chinese Medicine, Changsha, China; ^5^ Department of Endocrinology, The First Hospital of Hunan University of Chinese Medicine, Changsha, China

**Keywords:** diabetic nephropathy, Bu-Shen-Huo-Xue decoction, podocyte epithelial-mesenchymal transition, Rac1, PAK1, p38MAPK

## Abstract

Diabetic nephropathy (DN), a leading cause of end-stage renal disease, is associated with high morbidity and mortality rates worldwide and the development of new drugs to treat DN is urgently required. Bu-Shen-Huo-Xue (BSHX) decoction is a traditional Chinese herbal formula, made according to traditional Chinese medicine (TCM) theory, and has been used clinically to treat DN. In the present study, we established a high-fat diet/streptozotocin-induced diabetic mouse model and treated the mice with BSHX decoction to verify its therapeutic effects *in vivo*. Ultraperformance liquid chromatography coupled with quadrupole time-of-flight mass spectrometry (UPLC-Q-TOF-MS) was applied to analyze the chemical composition and active compounds of BSHX decoction. Markers of podocyte epithelial-mesenchymal transition and the Rac1/PAK1/p38MAPK signaling pathway were evaluated to investigate the mechanism underlying function of BSHX decoction. BSHX decoction effectively alleviated diabetic symptoms, according to analysis of the renal function indicators, serum creatinine, blood urea nitrogen, serum uric acid, and urinary albumin excretion rate, as well as renal histopathology and ultrastructural pathology of DN mice. We identified 67 compounds, including 20 likely active compounds, in BSHX decoction. The podocyte markers, nephrin and podocin, were down-regulated, while the mesenchymal markers, α-SMA and FSP-1, were up-regulated in DN mouse kidney; however, the changes in these markers were reversed on treatment with BSHX decoction. GTP-Rac1 was markedly overexpressed in DN mice and its levels were significantly decreased in response to BSHX decoction. Similarly, levels of p-PAK1 and p-p38MAPK which indicate Rac1 activation, were reduced on treatment with BSHX decoction. Together, our data demonstrated that BSHX decoction ameliorated renal function and podocyte epithelial-mesenchymal transition via inhibiting Rac1/PAK1/p38MAPK signaling pathway in high-fat diet/streptozotocin-induced diabetic mice. Further, we generated a quality control standard and numerous potential active compounds from BSHX decoction for DN.

## Introduction

Diabetes is a group of metabolic diseases, mainly caused by defects in insulin secretion, insulin activity, or both (American Diabetes Association, 2013). The number of people suffering from diabetes is predicted to rise to 590 million by 2,035 worldwide, while approximately 5.1 million individuals died from diabetes and its complications in 2013 (Sen and Chakraborty, 2015). Diabetic nephropathy (DN) is a common complication of diabetes. Early stage of DN is characterized by microalbuminuria, which can gradually lead to massive proteinuria, a declining glomerular filtration rate, and elevated creatinine, and finally, renal failure (Alicic et al., 2017). Further, DN can not only lead to uremia, but also macrovascular complications, such as heart attacks and strokes, which can be fatal (Forbes and Cooper, 2013). Development of new drugs and treatments is urgently needed to control the progress of DN and reduce associated mortality. Dysfunction or injury of podocyte, key components of the glomerular filtration barrier, has been proposed as the core pathophysiology underlying DN (Torban et al., 2019). Epithelial-mesenchymal transition (EMT) is primary mechanisms of podocyte injury (Dai et al., 2017). During EMT, epithelial cells lose their hallmark epithelial characteristics and gain the features of mesenchymal cells; this process can be regulated by several signaling pathways (Ying and Wu, 2017).

In traditional Chinese medicine (TCM), DN belongs to the category of “Xiaoke,” and the pathogenic features of “deficiency” and “stasis” are considered of great importance in the process of DN. Therapies involving “tonifying deficiency” and “invigorate the circulation of blood” should be adopted in TCM clinical practice (Fang et al., 2016). Bu-Shen-Huo-Xue (BSHX) decoction, from ancient Chinese literature “Shangke Dacheng,” is a traditional Chinese herbal formula composed of 11 individual Chinese herbs, according to TCM theory. Its components can be divided into two groups, one of which is used for “tonifying deficiency,” comprises *Psoraleae Fructus, Eucommia Cortex, Lycii Fructus, Cistanches Herba, Rehmanniae Radix Praeparata, Cuscutae Semen,* and *Corni Fructus*; and the other to “invigorate the circulation of blood,” comprising *Angelica Sinensis Radix, Angelicae Pubescentis Radix, Carthami Flos,* and *Myrrh*. Many herbs used in BSHX decoction have been demonstrated to relieve the symptoms of DN; for example, extraction of *Psoraleae Fructus* can significantly reverse increased creatinine clearance, urine volume, urine microalbumin, and mesangial expansion in streptozotocin (STZ)-induced diabetic mice (Seo et al., 2017). Our previous study confirmed that BSHX decoction can alleviate the proliferation inhibition, apoptosis, migration, and occurrence of EMT in podocytes that are induced in response to high glucose *in vitro* (Hu et al., 2019); however, whether it has a similar effect *in vivo* and the underlying mechanisms involved remain unclear. As Chinese herbal remedies often comprise multiple components, the main compounds present in BSHX decoction and which of them can be absorbed into the blood and have the potential to exert therapeutic effects, is also of interest.

Traditional Chinese herbal formulae generally contain numerous compounds; consequently, data on their pharmacokinetics properties, mechanisms of action, and safety profiles are frequently limited. Therefore, determination of the constituent compounds of such medicines, understanding the chemical basis of their activities, and establishment of quality control procedures have become a focus of intensive research, raising particular challenges (Zhang et al., 2016). Serum pharmacochemistry of traditional Chinese herbs is a method used to screen for effective substances by identification and analysis of constituents migrating to the blood after oral administration (Ma et al., 2017), since those herbal compounds that are absorbed into the circulation can be considered potential bioactive compounds associated with therapeutic effects. The development of Ultraperformance liquid chromatography coupled with quadrupole time-of-flight mass spectrometry (UPLC-Q-TOF-MS) has provided the technical means to study the multiple components of traditional Chinese herbs and their effective ingredients.

In this study, we comprehensively explore the effects of BSHX decoction *in vivo*, its chemical constituents and active compounds, and the mechanisms underlying its efficacy in treating DN. First, a high-fat diet (HFD)/STZ-induced DN mouse model was established and administered with BSHX decoction to examine its therapeutic effect. Second, based on the therapeutic effects of BSHX decoction, UPLC-Q-TOF-MS was applied to identify its chemical constituents and analyze its active compounds in medicated serum. Then, the markers of podocyte EMT and Rac1/PAK1/p38MAPK signaling were evaluated, to assess the mechanism underlying the effects of BSHX decoction.

## Materials and methods

### Compositions and Preparation of Bu-Shen-Huo-Xue Decoction

The Chinese herbs used in BSHX decoction are presented in Table 1. All herbs were provided by The First Hospital of Hunan University of Chinese Medicine and were identified by Professor Shaogui Liu. The authenticated samples were kept at the Experiment Center of Medical Innovation of The First Hospital of Hunan University of Chinese Medicine. First, BSHX decoction herbs were mixed and soaked for 1 h in 10 volumes of water (volume/weight), then extracted by refluxing for 1.5 h. The extracted solution was filtered and the extraction procedure repeated once. Second, the two extracted solutions combined were concentrated to a relative density of 1.70 g/ml. A medium dose of BSHX decoction was administered, corresponding to the clinical dose, which was calculated by conversion, based on the ratio of human to mouse (the human body weight was set as 60 kg).

**TABLE 1:** Chinese herbs of BSHX decoction.

Chinese name	English name	Latin name	Palace of Origin	Quantities (g)
Bu Gu Zhi	Psoraleae Fructus	*Cullen corylifolium (L.) Medik.*	Sichuan, China	15
Dang Gui	Angelica Sinensis Radix	*Angelica sinensis (Oliv.) Diels*	Gansu, China	10
Du Huo	Angelicae Pubescentis Radix	*Angelica biserrata (R.H.Shan and C.Q.Yuan) C.Q.Yuan and R.H.Shan*	Hubei, China	10
Du Zhong	Eucommia Cortex	*Eucommia ulmoides Oliv.*	Hunan, China	10
Gou Qi Zi	Lycii Fructus	*Lycium barbarum L.*	Ningxia, China	10
Hong Hua	Carthami Flos	*Carthamus tinctorius L.*	Xinjiang, China	6
Mo Yao	Myrrh	*Commiphora myrrha (T.Nees) Engl.*	Hainan, China	10
Rou Cong Rong	Cistanches Herba	*Cistanche deserticola Ma*	Neimenggu, China	10
Shu Di Huang	Rehmanniae Radix Praeparata	*Rehmannia glutinosa (Gaertn.) DC.*	Henan, China	15
Tu Si Zi	Cuscutae Semen	*Cuscuta chinensis Lam.*	Neimenggu, China	10
Shan Zhu Yu	Corni Fructus	*Cornus officinalis Siebold and Zucc.*	Henan, China	10

### Reagents

Serum creatinine (SCr) (lot#: C011-1-1), blood urea nitrogen (BUN) (#C013-1-1), serum uric acid (SUA) (#C012-1-1), triglyceride (TG) (#A110-1-1), total cholesterol (TC) (#A111-1-1), and urine protein (#C035-2-1) assay kits were from Nanjing Jiancheng Bioengineering Institute (Nanjing, China). Anti-α-smooth muscle actin (αSMA) antibody (#bs-0189R) was purchased from Beijing Biosynthesis Biotechnology Co.,Ltd. (Beijing, China). Anti-Rac1(#ab33186), anti-GTP-Rac1 (#ab33186), anti-p-PAK1 (#ab75599), anti-p38MAPK (#ab195049), anti-p-p38MAPK (#ab47363), anti-β-catenin (#ab32572), anti-Podocin (#ab50339), and anti-nephrin (#ab216341) antibodies were from Abcam (Cambridge, United Kingdom). Anti-β-actin (#66009-1-Ig), anti-fibroblast-specific protein-1 (FSP-1) (#16105-1-AP), anti-PAK1 (#21401-1-AP), anti-snail (#13099-1-AP), HRP goat anti-mouse IgG (#SA00001-1), and HRP goat anti-rabbit IgG (#SA00001-2) antibodies were from Proteintech (Chicago, United States). Trizol (#15596026) was from Thermo Scientific (MA, United States). A Reverse Transcription kit (#CW2569) was purchased from CWBio Co., Ltd. (Beijing, China). STZ (#WXBC8740V) was from Sigma-Aldrich Co. Ltd. (MO, United States). Metformin (#A181224) from Zhejian Yatai Pharmaceutical Co., Ltd. (Shaoxing, China).

### Animals

Eight-week-old male C57BL/6 mice were purchased from Beijing Vital River Laboratory Animal Technologies Co. Ltd (Animal license No.: 1100111911001622.) Animals were raised under specific pathogen-free conditions [certification No.: SCXK (Xiang) 2015-0003] at 22°C ± 1°C and humidity of 50 ± 5%, with a 12 h light/dark cycle. All animal experiments were conducted in accordance with the National Institutes of Health (NIH) Guide for the Care and use of Laboratory Animals and were approved by the Ethics Committee for Experimental Animals of The First Hospital of Hunan University of Chinese Medicine (Approval No.: ZYFY20181201).

### Animal Model and Experimental Groups

Mice (*n* = 60) were randomly divided into two groups: normal groups (NC, *n* = 10) and disease model (*n* = 50). The model group were fed with a HFD (HFD: 20% protein, 35% carbohydrate, and 45% fat; total calorie content, 4.73 kcal/gm) and the NC group were fed with a low-fat control diet (20% protein, 70% carbohydrate, 10% fat; total calorie content, 3.85 kcal/gm) for 4 weeks. In the fifth week, STZ (50 mg/kg) was injected intraperitoneally into model group mice for five consecutive days, whlie those in the NC group were injected with vehicle; mice were fasted for 8 h before injection, and 4 h after injection, return to the previous diet (Furman, 2015; Song et al., 2018). Fasting blood glucose (FBG) levels were measured 72 h after the last injection of STZ and only mice with FBG > 16.7 mM were included in the study, and randomly divided into five groups: 1) DN group; 2) BSHX decoction low dose treated group (DN + BSHX-L; 8.5 g/kg/d); 3) BSHX decoction medium dose treated group (DN + BSHX-M; 17.0 g/kg/d); 4) BSHX decoction high dose treated group (DN + BSHX-H; 34.0 g/kg/d); 5) metformin treated group (DN + MET; 0.1 g/kg/d). The NC and untreated DN groups were treated with the same amount of distilled water.

The body weight and FBG were measured every 2 weeks for 8 weeks in all mice following treatment. Urine samples were collected during the final week of treatment using a metabolic cage, and 24 h food intake, 24 h water intake, and 24 h urine volume measured. Mice were then sacrificed, their blood collected and centrifuged to obtain serum, and their kidneys removed and weighted. Urine and serum samples were rapidly transferred to −80°C for storage until use. Half of the right kidney was stored in 4% paraformaldehyde for hematoxylin/eosin (HE) and periodic acid-Schiff (PAS) staining, and immunohistochemical detection, and the other half in 2.5% glutaraldehyde for observation using a transmission electron microscope. Left kidneys were placed in cryotubes, snap frozen in liquid nitrogen, then stored at −80°C for western blotting and Real time quantitative PCR detection assays.

### Serum and Urine Biochemistry Assays

Mouse blood glucose concentration was tested using a Baianjin glucometer and test paper (Ascensia Diabetes Care Holdings AG, Shanghai, China). Scr, BUN, SUA, serum total cholesterol (TC), serum triglyceride (TC), and urinary albumin concentrations were all measured using commercial kits (Nanjing Jiancheng Bioengineering Institute Nanjing, China). All the tests were performed strictly according to the instructions provided by the manufacturers.

### Hematoxylin and Eosin and Periodic Acid-Schiff Staining

Kidney tissue specimens were fixed in 10% neutral formalin for 1–2 h, then dehydrated, immersed in wax, and sectioned. Sections were incubated at 60°C for 1–2 h, then placed in xylene for 10 min twice, followed by 100, 100, 95, 85, and 75% ethanol successively for 5 min each, then soaked in distilled water for 5 min.

#### Hematoxylin and Eosin Staining

Sections were then stained with hematoxylin for 5–10 min, then washed with distilled water, followed by destaining with PBS. Next, sections were stained with eosin for 3–5 min, washed with distilled water, dehydrated with alcohol, placed in xylene twice for 10 min, sealed with neutral gum and observed under a microscope.

#### Periodic Acid-Schiff staining

For PAS staining, 50 µl of iodate was added to cover the tissue, and allowed to rest for 5 min, washed for 10 min, dyed with Schiff’s solution for 5 min, then hematoxylin for 20 s, before washing with distilled water and destained with PBS, and drying. Subsequent dehydration and observation processes were the same as those used for HE staining.

### Immunohistochemistry staining

Paraffin-embedded kidney tissue sections were heated and dewaxed as described for HE staining. Then, sections were soaked in 0.01 M citrate buffer (pH 6.0) and boiled continuously for 20 min, before washing three times for 3 min in 0.01 M PBS (pH 7.2–7.6) following cooling for antigen repair. Diluted (1:200) primary antibodies (against podocin, nephrin, αSMA, or FSP-1) were added and specimens incubated overnight at 4°C, then rinsed three times for 5 min each. Next, samples were incubated with secondary antibody (50–100 L of anti-rabbit, rabbit, rabbit, rabbit-IgG antibody, or HRP polymer was added, followed by incubation at 37°C for 30 min. DAB working solution (50–100 μl) was added, samples incubated at room temperature for 1–5 min, and reaction time controlled by observation under a microscope. Next, samples were dyed using hematoxylin (5–10 min), and washed with distilled water and destained with PBS, followed by dehydration with alcohol, and placing in xylene for 10 min twice, sealing with neutral gum, and observation under a microscope. Yellow or brown-yellow (dark to brown) staining was considered positive.

### Observation by Transmission Electron Microscopy

After fixing in 2.5% glutaraldehyde for 6–12 h, renal tissue samples were placed in PBS buffer for 1–6 h, then transferred to osmium acid for 1–2 h. Samples were then dehydrated using a graded alcohol series (30, 50, 70, 80, 95, and 100%) and propylene epoxide, soaked in propylene epoxide and epoxy resin (1:1) for 1–2 h, and then pure epoxy resin for 2–3 h. After embedding in pure epoxy resin, samples were baked in an oven at 40°C for 12 h and sectioned into ultra-thin slices. Following electron staining with lead and uranium, samples were imaged using a transmission electron microscope (HITACHI 7700) and images recorded using a digital camera.

### Analysis of Bu-Shen-Huo-Xue Decoction Chemical Ingredients and its Serum Pharmacochemistry Using Ultraperformance Liquid Chromatography Coupled with Quadrupole Time-of-Flight Mass Spectrometry Technology

#### Reagents

Hydroxysafflor yellow A (CAS No.: 78281-02-4, lot#: 111637-201810), Neobavaisoflavone (41060-15-5, #520052-201401), and Osthole (484-12-8, #110822-201710) were purchased from the National Institutes for Food and Drug Control (Beijing, China). Loganin (18524-94-2, #L2282EA34), Hyperoside (482-36-0, #Q6112C185) were from ACMEC biochemical Co., Ltd (Shanghai, China); Psoralen (66-97-7, #191201-018), Angelicin (523-50-2, #190726-004), Angelol A (19625-17-3, #191211-200), and Angelol G (83199-38-6, #191124-199) were from Beijing Zhongxing Jiaren Biotechnology Co., Ltd (Beijing, China).

#### Ultraperformance Liquid Chromatography Coupled with Quadrupole Time-of-Flight Mass Spectrometry Assay

First, test BSHX decoction sample, medicated and non-medicated serum, and standard substances were prepared.

##### Bu-Shen-Huo-Xue Decoction

BSHX decoction extract (2.000 g) was accurately weighed and methanol added to a final volume of 10.00 ml, which was then treated using ultrasonic waves for 30 min and filtered through a microporous membrane (0.22 μm) as the testing sample.

##### Standard Substances

Nine standard substances were weighed (10.00 mg each) of and methanol added to dissolve and dilute them in a final volume of 25.00 ml. Then, 100 μl aliquots were taken from each of the nine solutions and mixed as test samples.

##### Medicated and Non-Medicated Serum

Blood was collected from mice 60 min after the last treatment to prepare medicated and non-medicated serum. Then, serum samples were centrifuged at 7,000 × *g* for 5 min, and 2 ml taken and added to three volumes of methanol. After rotating for 10 min and one more centrifugation, sample supernatants were volatilized until dry, then redissolved in 100 ml methanol and centrifuged at 12000 rpm for 10 min. Next, samples were filtered through microporous membranes (0.22 μm) to obtain medicated and non-medicated serum samples.

##### Ultraperformance Liquid Chromatography Coupled with Quadrupole Time-of-Flight Mass Spectrometry

UPLC-Q-TOF-MS (1290 UPLC-6540, Agilent Technologies Inc., United States) was used to analyze the prepared samples. Chromatographic conditions were as follows: An Agilent ZORBAX Eclipse Plus C18 (3.0 × 100 mm, 1.8 μm) column was used and the mobile phase system was composed of acetonitrile (A) and water (contained 0.1% formic acid). A gradient elution procedure was used, as follows: 0–10 min, 5–15% A; 10–15 min, 15–20% A; 15–25 min, 25–45% A; 25–40 min, 45–80%; flow velocity, 0.4 ml/min; and the sample volume, 1 μl. Mass spectrometry testing conditions were as follows: ionization mode, electrospray ionization and the accurate mass data correction using electrospray ionization-L Low Concentration Tuning Mix (G1969-85000). Positive and negative ion analysis mode and MRE scan mode were adopted. The range of full-mass scanning was 100–1700 m/z. Sheath gas temperature was 350°C and the capillary voltage of 4.0 KV. The desolventize gas was nitrogen, and the temperature of the dry gas was 325°C, with a flow rate of 6.8 L/min. Secondary mass spectrometry was performed by dependent scanning, and the first three strengths were selected for collision induced dissociation (CID), based on of primary scanning to obtain secondary mass spectrometry data. The secondary fragment scan range was 50–1000 and fragment voltages were 10, 20, and 30 KV.

### Compounds Identification

Compounds were identified by extracting ion flow diagrams and comparing retention times with those of relevant reference materials, or by predicting the molecular formulae of compounds from their accurate relative molecular weights, combined with information collected from the literature and databases. Subsequently, compounds that were absorbed into the blood were ascertained by comparing the chemical composition of BSHX decoction, medicated serum, and non-medicated serum. Using the concentration and peak areas of standard substances, some compounds were identified and quantified according to the external standard method. The calculation formula used was:

$$Cx=\frac{Ax}{As}\times Cs$$

Where Cx is the concentration of a compound to be calculated, Ax the peak area of a compounds to be calculated, Cs the concentration of a corresponding standard substance, and As the peak area of a corresponding standard substance.

### Western Blot Analysis

The GTP-Rac1 protein extraction and its detection were conducted according to the manufacturer’s instructions for the Active Rac1 Pull-Down and Detection Kit (Thermo Scientific, Massachusetts, United States, lot#: UJ296831). Proteins were extracted by adding RIPA lysis buffer to kidney tissue, followed by repeated grinding, and incubation on ice for 10 min, and centrifugation at 16000 × *g* for 15 min at 4°C to obtain supernatant. For western blot, 240 μl of protein supernatant was added to 60 μl 5× loading buffer, mixed, boiled for 5 min, then cooled on ice. Tissue lysate samples were resolved by SDS-PAGE, then transferred to nitrocellulose membranes. PBST (1×) was used to prepare 5% defatted milk powder, in which membranes were immersed at room temperature for 90 min. Membranes were then incubated at 4°C overnight with primary antibody diluted in 1× PBST as follows: anti-Rac1, anti-GTP-Rac1, anti-p38MAPK, anti-podocin, anti-nephrin, anti-FSP-1, and anti-α-SMA, 1:1,000; anti-p-PAK1, anti-p-p38MAPK, and anti-snail, 1:750; anti-PAK1, 1:2,500; anti-β-catenin, 1:7,500; β-actin, 1:5,000. The next day, membranes were incubated at room temperature for 30 min in HRP-labeled secondary antibody diluted in 1× PBST as follows: HRP goat anti-mouse IgG, 1:5,000; and HRP goat anti-rabbit IgG, 1:6,000. Membranes were then incubated at room temperature for 90 min, followed by three washes, and addition of ECL chemiluminescent solution for color development. Exposed films were scanned and analyzed using Quantity One software.

### Real Time Quantitative PCR

Total RNA was extracted from animal tissues using Trizol reagent and the integrity of RNA confirmed by agarose gel electrophoresis. cDNA was reverse-transcribed from mRNA using a reverse transcription kit. RT-qPCR was performed using SYBR green assays. The sequences of target genes were searched from the NCBI database, and primer5 software was used to design primers. The primer sequences were as follows: *Rac1*: 5ʹ-CGT​CCC​CTC​TCC​TAC​CCG​CAG​A-3ʹ (forward) and 5ʹ-TGT​CGC​ACT​TCA​GGA​TAC​CAC​T-3ʹ (reverse), product length 164 bp; *Pak1*: 5ʹ-AAA​CCT​CTG​CCT​CCA​AAC​CC-3ʹ (forward) and 5ʹ-CAC​TGT​TCT​GGC​ATT​CCC​GTA-3ʹ (reverse), product length 194 bp; *p38Mapk*: 5ʹ-CTC​ATT​AAC​AGG​ATG​CCA​AGC​C-3ʹ (forward) and 5ʹ-AGC​ATC​TTC​TCC​AGT​AGG​TCG-3ʹ (reverse), product length 131 bp; *Snail*: 5ʹ-TGC​TTT​TGC​TGA​CCG​CTC​CAA​C-3ʹ (forward) and 5ʹ-GCA​CTG​GTA​TCT​CTT​CAC​ATC​CGA​GT-3ʹ (reverse), product length 70 bp; *β-Catenin*: 5ʹ-CAG​TCC​TTC​ACG​CAA​GAG​C-3ʹ (forward) and 5ʹ-ATG​CCC​TCA​TCT​AGC​GTC​T-3ʹ (reverse), product length 107 bp; actin: 5ʹ-ACA​TCC​GTA​AAG​ACC​TCT​ATG​CC-3ʹ (forward) and 5ʹ-TAC​TCC​TGC​TTG​CTG​ATC​CAC-3ʹ (reverse), product length 223 bp. The 2^−ΔΔCt^ method was used to analyze relative gene expression levels.

### Statistical Analysis

Statistical analyses were performed using SPSS25.0 software. All data were presented as mean ± standard error of mean (SEM). One-way ANOVA or two-way repeated-measures ANOVA were used to compare differences among three or more groups, and post-hoc Fisher’s least significant difference (LSD) test or Dunnett’s test for the individual group comparisons. Values of *p* < 0.05 were considered statistically significant.

## Results

### Bu-Shen-Huo-Xue Decoction Reduces Diabetic Metabolic Parameters and Urinary Albumin Excretion, and Ameliorates Impaired Kidney Function in Diabetic nephropathy Mice

**FIGURE 1:**
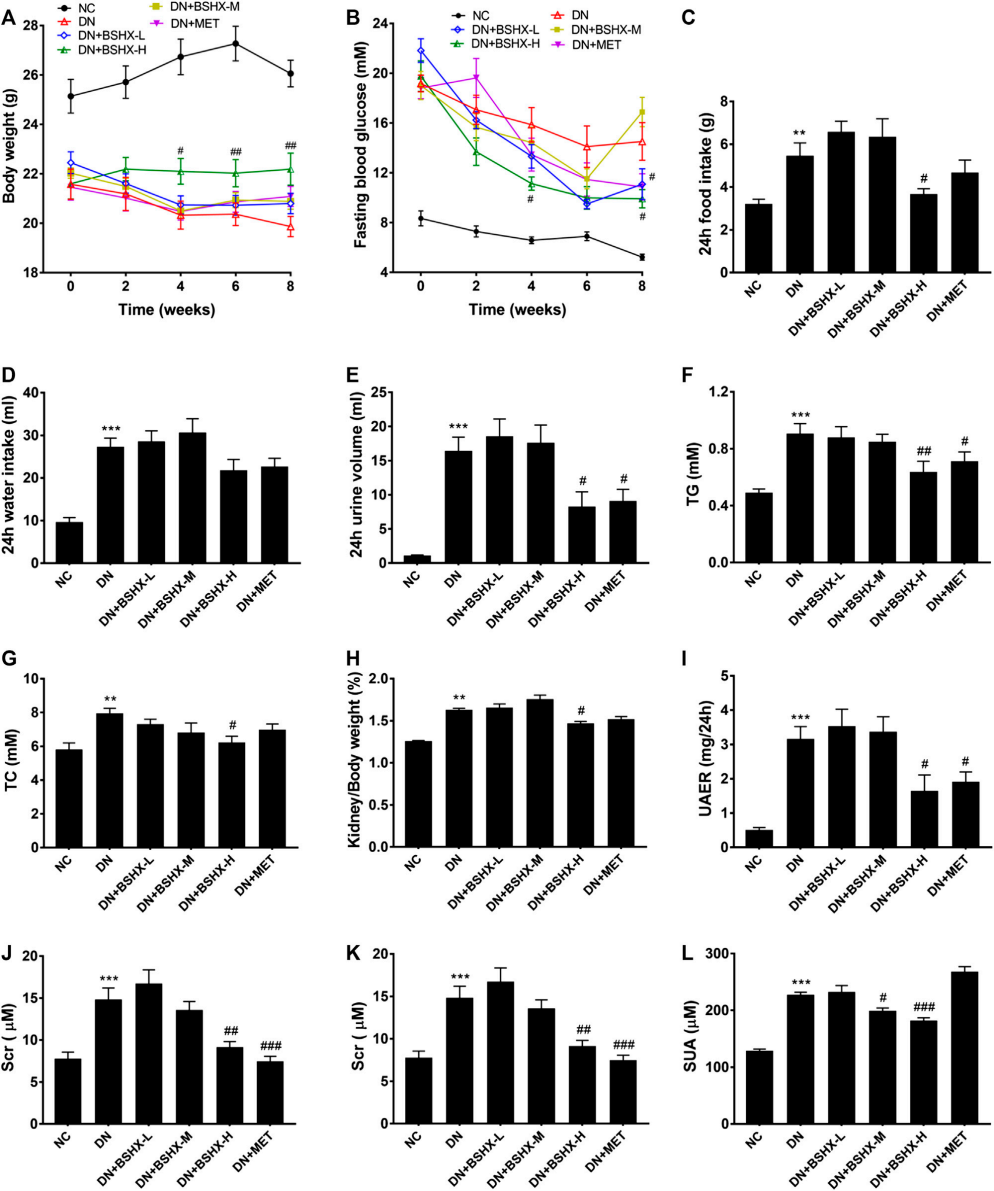
BSHX decoction reduces diabetic metabolic parameters, urinary albumin excretion and ameliorates kidney function in DN mice. **(A)** Changes in body weight. **(B)** Changes in fasting blood glucose. **(C)** 24 h food intake. **(D)** 24 h water intake. **(E)** 24 h urine volume. **(F)** Serum triglyceride (TG). **(G)** Serum total cholesterol (TC). **(H)** Kidney weight/body weight. **(I)** Urinary albumin excretion rate (UAER). **(J)** Serum creatinine (SCr). **(K)** Blood urea nitrogen (BUN). **(** **L)** Serum uric acid (SUA). *n* = 5–9 mice per group. All data are expressed as means ± SEM. ^*^ *p* < 0.05, ^*^^*^ *p* < 0.01, ^*^^*^^*^ *p* < 0.001 vs. the NC group; ^#^ *p* < 0.05, ^##^ *p* < 0.05, ^###^ *p* < 0.001 vs. DN model group.

As shown in Figures 1A, the body weight of DN model mice were gradually decreased compared with that of NC group; however, mice treated with high dose BSHX decoction, maintained a relatively stable body weight, which differed significantly from the DN group at week 4 after treatment. In addition, the other groups were also improved at week 4, but did not differ significantly compare with the DN group until week 8. FBG showed a decreased trend in DN mice; however, it decreased more significantly and more smoothly after treatment with high dose BSHX decoction and metformin (Figures 1B). Using a metabolic cage, 24 h food intake, 24 h water intake, and 24 h urine volume, which were significantly increased in DN group mice, were calculated during the eight week after treatment. As shown in Figures 1C–E 24 h food intake and 24 h urine volume were clearly decreased in response to treatment with high dose BSHX decoction and metformin. Interestingly, there was no significant difference of 24 h water intake, although there was a trend consistent with food intake and urine volume. Further, there were no significant differences of food intake, water intake, or urine volume in mice treated with low or medium dose BSHX decoction. Additionally, we found that treatment with BSHX decoction significantly lowered serum TG and TC levels, which were increased in the DN group, in a dose-dependent manner (Figures 1F,G). Moreover, the kidney weight/body weight reduced after administration of high dose BSHX decoction for 4 weeks; however, there was no significant difference between the metformin-treated and DN model groups, although a decreasing trend was detected (Figures 1H).

DN is a disease characterized by massive proteinuria and impaired renal function. Our data showed that albuminuria and renal function indicators of DN mice were significantly increased compared with the NC group; however, after 8 weeks treatment with metformin or BSHX decoction, there was not only a significant decrease in urinary albumin excretion rate (UAER) in a relatively dose-dependent manner compared to the DN model group, but SCr, BUN, and SUA as well (Figures 1I–L). The increase in SUA observed in the metformin-treated group was unexpected, and led us to suspect that there was a deviation existed in some process, such as serum sample storage or treating process, or determine process, fortunately, this deviation has not impaired the integrity of the data.

### Bu-Shen-Huo-Xue Decoction Alleviates Histopathology and Ultrastructural Pathology of the Kidney

**FIGURE 2:**
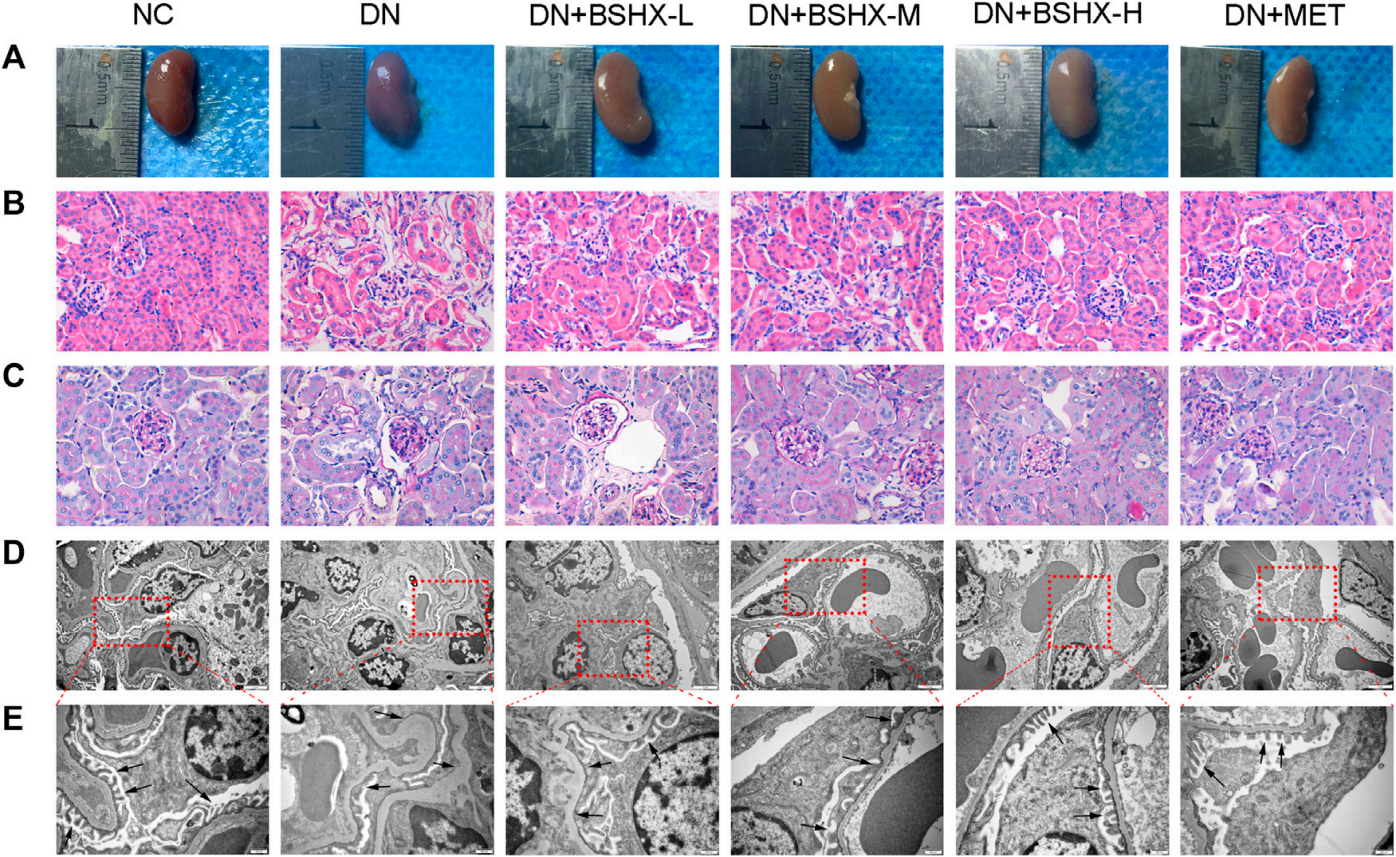
BSHX alleviates renal histopathology and ultrastructural pathology of the kidneys. **(A)** Preliminary observation of the appearance and size of kidneys. **(B)** Hematoxylin and eosin (HE) staining of the kidney (×400). **(C)** Periodic acid Schiff (PAS) staining of the kidney (×400). **(D)** Transmission electron microscopy of the kidney (scale bar, 2 μm). **(E)** Transmission electron microscopy of the kidney (scale bar, 500 nm). *n* = 5 mice per group.

First, we determined kidney size and weighted in the different experimental groups of mice. Figures 2A shows a hypertrophic kidney from a DN model group mouse compared with one from the NC group. Hypertrophy was relieved by administration of BSHX decoction and metformin. Second, we conducted HE and PAS staining of kidney samples. In contrast to the NC group, HE staining showed that glomerular volume was enlarged in DN group mice, and the number of cells was increased, mesangial matrix proliferated, cells around the glomerulus were disordered, and there was edema (Figures 2B). PAS staining showed glomerular hypertrophy, basement membrane thickening, and increased glycogen in the DN model group (Figures 2C). Changes detected by both HE and PAS staining in the DN model group were alleviated by the treatment of BSHX decoction, particularly at a high dose, and metformin. Furthermore, Transmission electron microscopy was used to observe the ultrastructural changes in the basement membrane and podocyte foot processes. This analysis revealed podocyte fusion and glomerular basement membrane thickening in the model group, and these pathological changes were ameliorated by treatment with BSHX decoction and metformin (Figures 2D,E).

### Chemical Composition and Serum Pharmacochemistry Analysis of Bu-Shen-Huo-Xue Decoction

**FIGURE 3:**
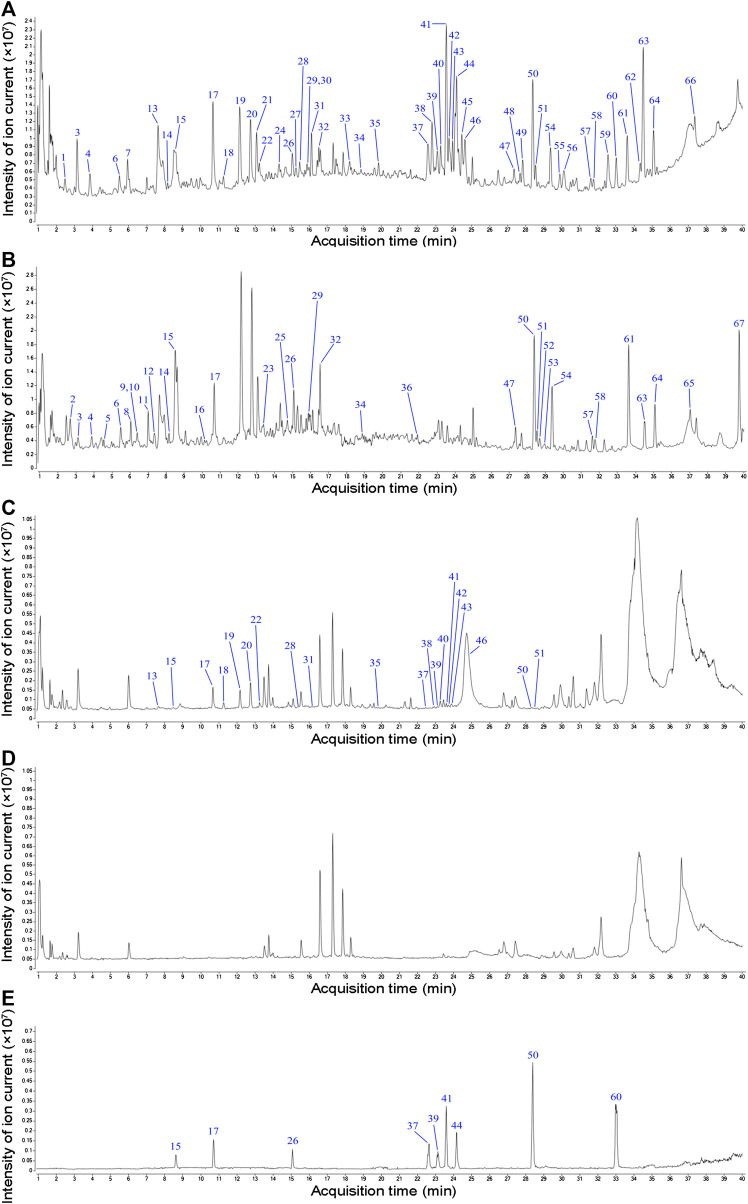
Total ion chromatograms (TIC) from BSHX decoction, medicated serum, non-medicated serum, and standard substances. **(A)** TIC of BSHX decoction in positive mode. **(B)** TIC of BSHX decoction in negative mode. **(C)** TIC of medicated serum in positive mode. **(D)** TIC of non-medicated serum in positive mode. **(E)** TIC of standard substances. Peak numbers correspond to compound numbers (see Supplementary Table S2).

Next, we evaluated the chemical composition of BSHX decoction to determine how it exerts its therapeutic effects on diabetic mice using positive and negative ion mode UPLC-Q-TOF-MS. Total ion chromatograms (TIC) of BSHX decoction, medicated serum, non-medicated serum, and standard substances are presented in Figure 3. Through analysis, 67 compounds of BSHX decoction and 20 compounds absorbed into the blood has been identified and its information of retention time, compound name, formula, error and its source were presented at Supplementary Table S2. The 67 BSHX decoction compounds identified included nine organic acids, 22 flavonoids, 15 glycosides, 13 terpenoids, six ester compounds, and two coumarins, most which were derived from the herbs *Psoraleae Fructus* (16 compounds) *Angelicae Pubescentis Radix* (15 compounds), and *Eucommia Cortex* (12 compounds).

**FIGURE 4:**
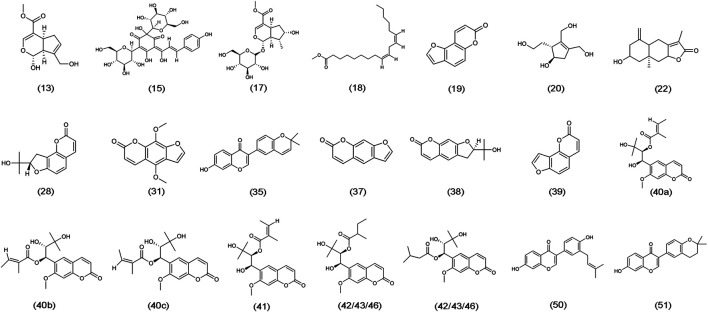
Chemical structure formulae of 20 compounds absorbed into the blood. Chemical structures and formulae of 20 compounds from PubChem (https://pubchem.ncbi.nlm.nih.gov/), numbered according to the TIC presented in Figure 3 and compounds information in Supplementary Table S2.

**FIGURE 5:**
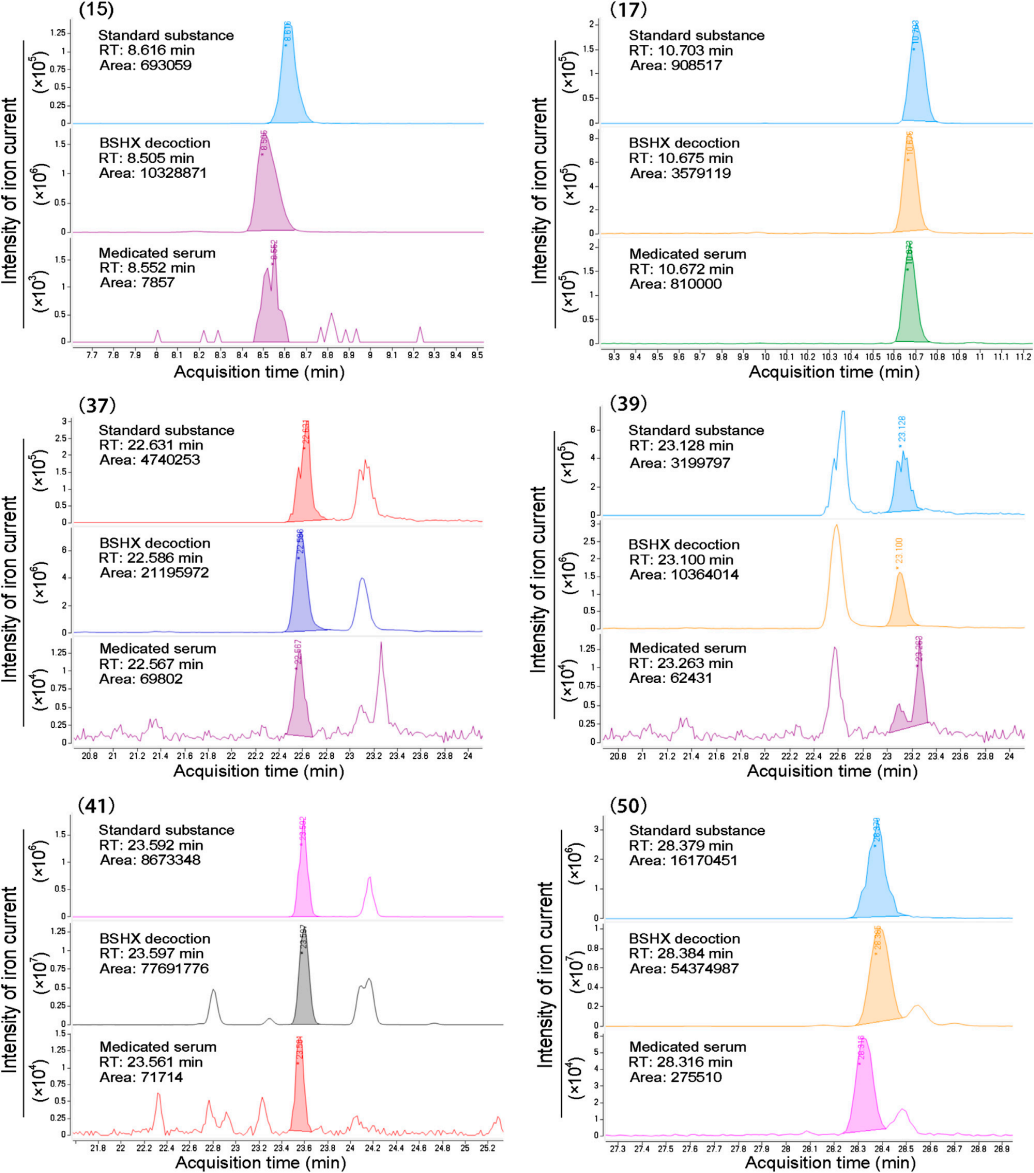
Peak areas information of six compounds determined using standard substances. Standard substance, BSHX decoction, and medicated serum samples peak of six compounds retention time (RT), area, and intensity of ion current, which cohere with the data presented in Table 2.

The chemical structure formulae of the 20 compounds identified in medicated serum, obtained from PubChem (https://pubchem.ncbi.nlm.nih.gov/) are present in Figure 4. Quantitative analysis of six compounds from BSHX decoction and medicated serum are shown in Table 2 and Figure 5. We found that the 20 compounds detected in medicated serum were from seven herbal constituents of BSHX decoction, as follows: *Eucommia Cortex, Carthami Flos, Lycii Fructus, Psoraleae Fructus, Myrrh, Angelicae Pubescentis Radix*, and *Corni Fructus*. Among them, A*ngelicae Pubescentis Radix* and *Psoraleae Fructus* had the highest number of compounds, at 8 and 5, respectively. Quantitative analysis showed that *Hydroxysafflor yellow A* and *Angelol A*, from *Carthami Flos* and *Angelica Sinensis Radix*, were present at the lowest concentrations (0.78 × 10^−2^ mM) in the medicated serum. Conversely, *Loganin*, from the herb, *Corni Fructus*, had the highest concentration (0.93 mM).

**TABLE 2:** Quantitative analysis information of six compounds in BSHX decoction and the medicated serum.

NO.	Compound (M + H^+^)	Determination of material	RT (min)	Area	Area%	Height	Max Y	Concentration (mM)
15	Hydroxysafflor yellow A (613.17)	Standard substance	8.616	693,059	100	137,604	138,846	0.69
		BSHX decoction	8.505	10,328,871	100	16,68,432	1,701,596	10.26
		Medicated serum	8.552	7,857	100	1,839	1,839	0.78 × 10^−2^
17	Loganin (391.10)	Standard substance	10.703	908,517	100	198,582	204,010	1.05
		BSHX decoction	10.675	3,579,119	100	819,828	845,941	4.13
		Medicated serum	10.672	810,000	100	205,680	210,103	0.93
37	Psoralen (187.03)	Standard substance	22.631	4,740,253	100	724,919	736,082	2.34
		BSHX decoction	22.586	21,195,972	100	2,956,094	2,999,738	10.44
		Medicated serum	22.567	69,802	100	11,838	12,812	0.34 × 10^−1^
39	Angelicin (187.03)	Standard substance	23.128	3,199,797	100	428,622	458,025	2.55
		BSHX decoction	23.100	10,364,014	100	1,539,838	1,627,915	8.26
		Medicated serum	23.263	62,431	100	11,571	13,976	0.50 × 10^−1^
41	Angelol A (377.16)	Standard substance	23.592	8,673,348	100	1,792,473	1,795,711	0.94
		BSHX decoction	23.597	7,7,691,776	100	13,144,727	13,214,665	8.43
		Medicated serum	23.561	71,714	100	13,888	14,512	0.78 × 10^−2^
50	Neobavaisoflavone (323.12)	Standard substance	28.379	16,170,451	100	3,269,979	3,333,621	3.51
		BSHX decoction	28.384	54,374,987	100	9,703,411	10,168,491	11.79
		Medicated serum	28.316	275,510	100	55,958	58,873	0.60 × 10^−1^

### Bu-Shen-Huo-Xue Decoction up-Regulates Nephrin and Podocin, and Down-Regulates Fibroblast-Specific Protein-1 and α-Smooth Muscle Actin, Expression in Diabetic nephropathy Mouse Kidneys

**FIGURE 6:**
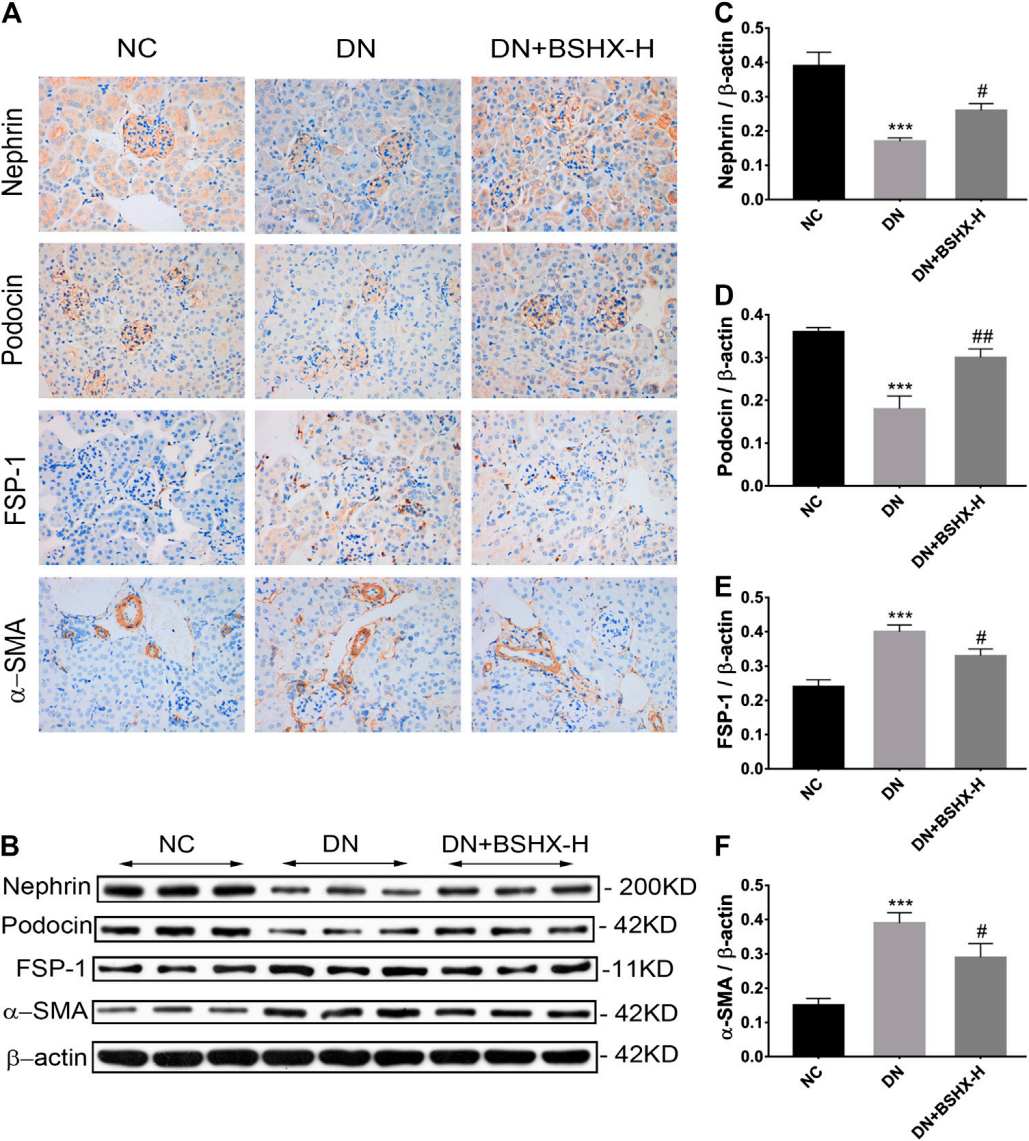
Effect of BSHX decoction on nephrin, podocin, FSP-1, and α-SMA levels in DN mouse kidney. **(A)** IHC staining of the kidney (×400) to analyze the expression levels of nephrin, podocin, FSP-1, and α-SMA. **(B)** Western blot assays to evaluate the expression levels of nephrin, podocin, FSP-1 and α-SMA. Beta-actin was measured as the loading control. **(C)** Quantitative analysis of the relative expression levels of nephrin. **(D)** Quantitative analysis of the relative expression levels of podocin. **(E)** Quantitative analysis of the relative expression levels of FSP-1. **(F)** Quantitative analysis of the relative expression levels of α-SMA. *n* = 5 mice per group. All data are expressed as means ± SEM. ^*^ *p* < 0.05, ^*^^*^ *p* < 0.01, ^*^^*^^*^ *p* < 0.001 vs. the NC group; ^#^ *p* < 0.05, ^##^ *p* < 0.05, ^###^ *p* < 0.001 vs. the DN model group.

Subsequent experiments focused on treatment with high dose BSHX (BSHX-H), based on our data demonstrating that it had the most significant effects on DN mice. Podocyte EMT is proposed to participate in podocytes injury, and is characterized by loss of epithelial cell markers, including nephrin and podocin, and gain of fibroblastic, such as markers-α-SMA and FSP-1 (Lv et al., 2013; Ying and Wu, 2017). Immunohistochemistry (IHC) staining showed that nephrin and podocin expression were decreased in DN mice and up-regulated in response to treatment with high dose BSHX decoction. In contrast, FSP-1 and α-SMA levels were increased in DN mice and down-regulated by high dose BSHX decoction (Figures 6A). To further confirm the effect of BSHX decoction on the expression of nephrin, podocin, FSP-1, and α-SMA, we conducted western blots, with results consistent with IHC staining (Figures 6B–F).

### Bu-Shen-Huo-Xue Decoction Inhibits the Rac1/PAK1/p38MAPK Signaling Pathway in Diabetic nephropathy Mouse Kidneys

**FIGURE 7:**
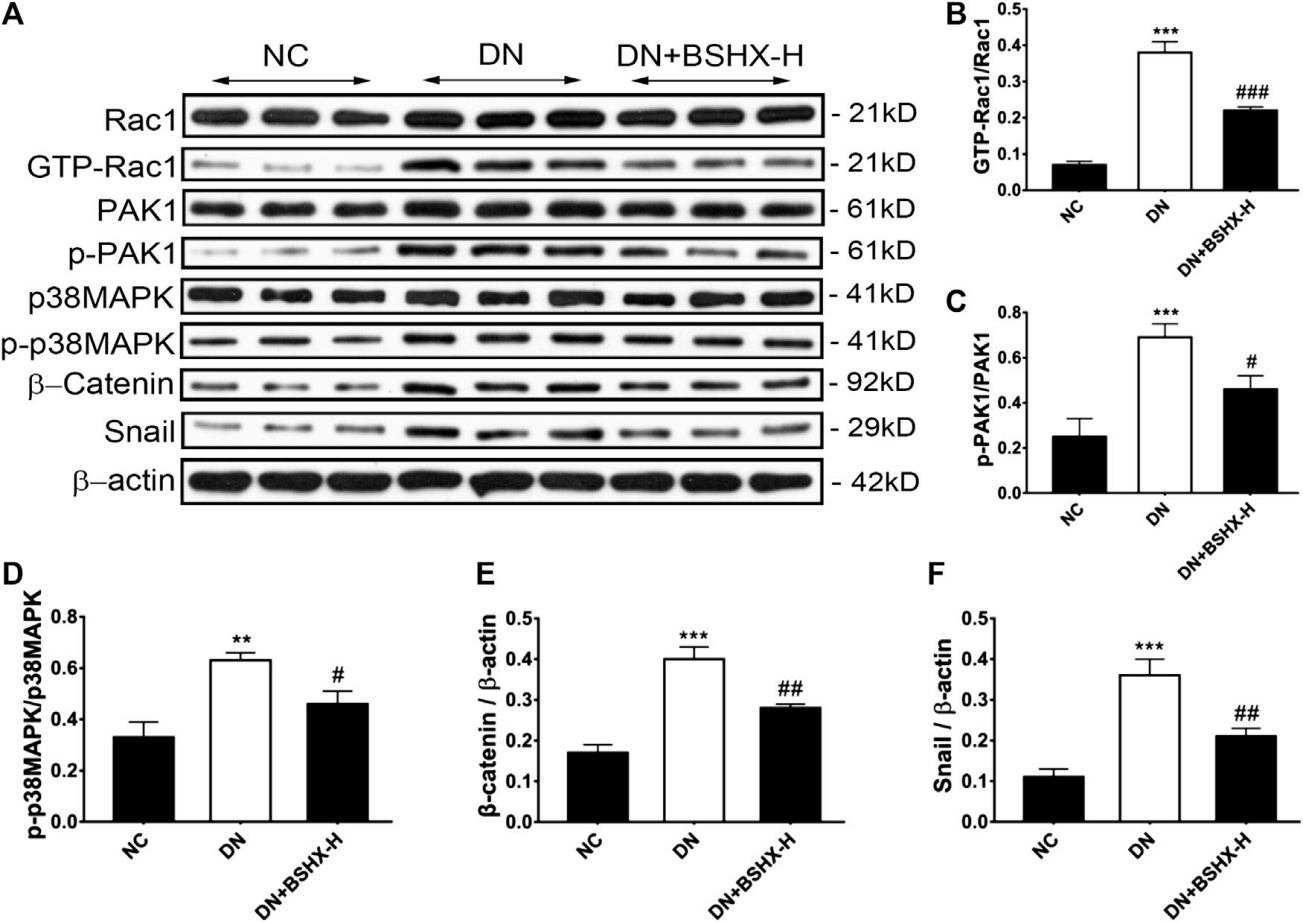
Effect of BSHX decoction on expression of Rac1/PAK1/p38MAPK signaling pathway proteins DN mouse kidney. **(A)** Representative western blots for Rac1, GTP-Rac1, PAK1, p-PAK1, p38MAPK, β-Catenin, and Snail protein expression in kidney tissue. β-actin was included as a loading control. **(B)** Quantitative analysis of GTP-Rac1/Rac1. **(C)** Quantitative analysis of p-PAK1/PAK1. **(D)** Quantitative analysis of p-p38MAPK/p38MAPK. **(E)** Quantitative analysis of Snail/β-actin. **(F)** Quantitative analysis of β-Catenin/β-actin. Band intensities were quantified by using Quantity One software. *n* = 5 mice per group. All data are expressed as means ± SEM. ^*^ *p* < 0.05, ^*^^*^ *p* < 0.01, ^*^^*^^*^ *p* < 0.001 vs. the NC group; ^#^ *p* < 0.05, ^##^ *p* < 0.05, ^###^ *p* < 0.001 vs. the DN model group.

**FIGURE 8:**
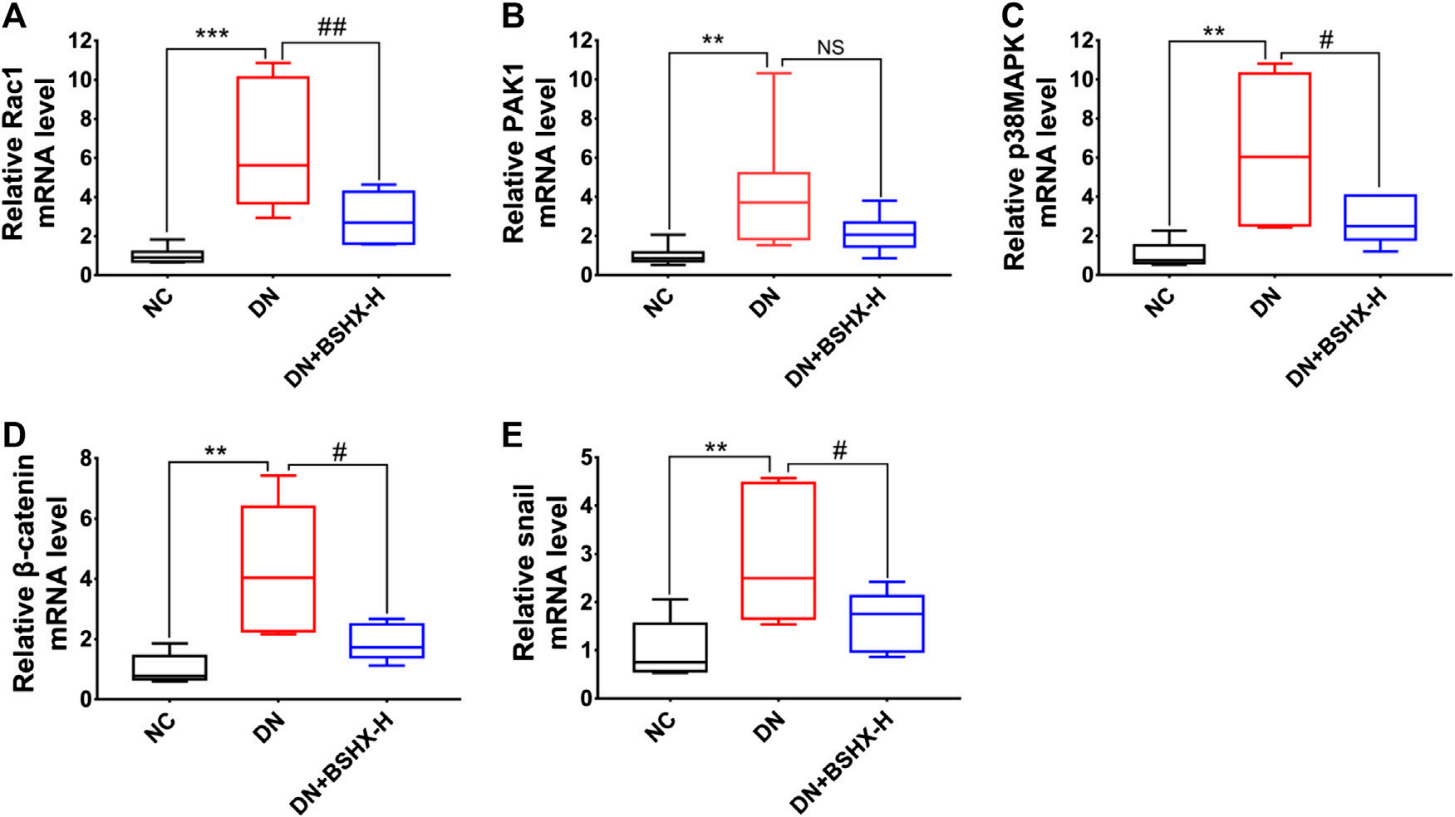
Effect of BSHX decoction on Rac1/PAK1/p38MAPK signaling pathway mRNA expression in DN mouse kidney. **(A)** Relative mRNA expression levels of *Rac1*. **(B)** Relative mRNA expression levels of *Pak1*. **(C)** Relative mRNA expression levels of *p38Mapk*. **(D)** Relative mRNA expression levels of *β-Catenin*. **(E)** Relative mRNA expression levels of *Snail*. The 2^−ΔΔCt^ method was used to analyze relative gene expression. *n* = 5 mice per group. All data are expressed as means ± SEM. ^*^ *p* < 0.05, ^*^^*^ *p* < 0.01, ^*^^*^^*^ *p* < 0.001 vs. the NC group; ^#^ *p* < 0.05, ^##^ *p* < 0.05, ^###^ *p* < 0.001 vs. the DN model group.

The Rac1/PAK1 axis is closely related to podocyte EMT, which may participate in deterioration of DN (Lv et al., 2013). Thus, we evaluated whether BSHX decoction influenced Rac1/PAK1/p38MAPK signaling to alleviate EMT of podocytes and exert its therapeutic effects in DN mice. Levels of Rac1, GTP-Rac1, PAK1, p-PAK1, p38MAPK, p-p38MAPK, β-Catenin, and Snail were detected by western blot or RT-qPCR. Western blot data are presented in Figure 7. Levels of GTP-Rac1/Rac1, p-PAK1/PAK1, p-p38MAPK/p38MAPK, β-Catenin/β-actin, and Snail/β-actin were notably up-regulated in DN mice and there was a significant difference following treatment with high dose BSHX decoction for 8 weeks. RT-qPCR data are presented in Figure 8. As it shown, *Rac1*, *Pak1*, *p38Mapk*, *β-Catenin* and *Snail* mRNA levels were significantly increased in DN mice and it was down-regulated after treatment with BSHX decoction. There was no significant difference in *Pak1* levels in the BSHX-H group compared with the DN model group that showed in Figures 8B, however, there was still a reducing trend of it.

**FIGURE 9:**
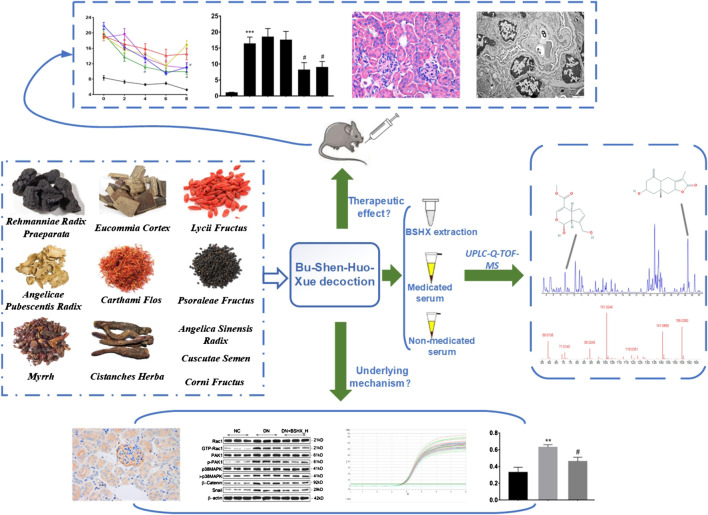
**Research conducted in this study.** This study was divided into three main parts: 1) verification of the therapeutic effects of BSHX decoction in DN mice; 2) ascertainment of the components and potential active compounds in BSHX decoction using UPLC-Q-TOF-MS; and 3) exploration of the mechanism underlying BSHX decoction activity using IHC, western blot, and RT-qPCR to detect podocyte EMT markers and factors involved in Rac1/PAK1/p38MAPK signaling.

## Discussion

DN is a diagnosis that refers to specific pathologic structural and functional changes in the kidneys of patients with both type 1 and type 2 diabetes mellitus (T1/T2DM), that results from the effects of DM on the kidney (Umanath and Lewis, 2018), and is a leading causes of end-stage renal disease (Sanajou et al., 2018). At present, the main treatment strategy for DN involves reducing proteinuria, preventing the progressive decline of glomerular filtration, and controlling related cardiovascular risk factors (Dong et al., 2016; Hung et al., 2017). Strictly controlling blood glucose levels and blood pressure, limiting protein intake, correcting lipid metabolic disorder, quitting smoking, and receiveing treatment with angiotensin-converting enzyme inhibitor, statins, antioxidants, and thiazolidinedione can control DN (Haghighatdoost et al., 2015; Pugliese et al., 2019); however, the incidence and disease progression rates remain relatively high in most patients with DN, and simply controlling blood glucose levels does not offer sufficient protection (Bilous, 2008). While the inhibition of the renin-angiotensin-aldosterone system may provide some benefits for patients with DN, many continue to develop end-stage renal disease (Xue et al., 2017). Thus, the challenges of exploring the mechanisms underlying DN and developing effective therapeutic methods or novel drugs to delay or prevent the progression of DN remain enormous for the foreseeable future. Attention casted to complementary and alternative therapies may provide us a more extensive research thought or latent resolvable approaches.

In China, TCM has an extensive history of application and there is a substantial literature on the treatment of DN. Furthermore, a variety of TCM extracts or formulae have been demonstrated have a certain renal protective effect (Wang et al., 2019b; Chen et al., 2019). In the theory of TCM, the location of DN is mainly in the kidney, and the pathogenic feature of “deficiency,” including qi, yin and kidney deficiency, and “stasis,” are considered to contribute to DN progression; hence, “tonifying deficiency” and “invigorating the circulation of blood” are the most crucial therapeutic measures for DN. Consistent with TCM theory, there is also abundant experimental evidence, supporting the clear potential of BSHX decoction for treatment of DN. In BSHX decoction, *Rehmanniae Radix Praeparata* can nourish the kidney and strengthen the essence, according to TCM theory clinical practice, and is commonly used to treat not only diabetes, but also kidney diseases such as nephrotic syndrome and chronic nephritis as well (Yu, 2019); *Rehmanniae Radix Praeparata* is also reported to decrease Scr and BUN, ameliorate glomerular sclerosis, and alleviate abnormal collagen distribution and interstitial fibrosis in rat model of renal interstitial fibrosis (Liu et al., 2015). *Corni Fructus,* a herb used to tonify the liver and kidney, is frequently used to treat internal heat and wasting-thirst, and is reported to reduce blood glucose and blood lipids, improving oxidative stress (Wang et al., 2019a), and protecting the kidney and β-cells (Lin et al., 2011; Gao et al., 2012) in diabetic mice. Similarly, extract of *Eucommia Cortex, Fructus Lycii* is reported to have significant antihyperglycemic and anti-inflammatory activities (Xie and Du, 2011; Zhang et al., 2015). The herbs in BSHX decoction mentioned above are all used for “tonifying deficiency.” On the other hand, *Angelica Sinensis Radix,* for tonifying and invigorating blood, exerts hypoglycemic and hypolipidemic benefits, potentially via ameliorating insulin resistance in STZ-induced diabetic BALB/c mice (Wang et al., 2015). *Carthami Flos,* which activates blood and resolves stasis, can reduce fat mass and glucose levels, as well as improving insulin sensitivity in HFD-induced obese mice (Yan et al., 2020). *Myrrh* is also reported have a kidney protective effect by up-regulating Nrf2/ARE/HO-1 signaling (Mahmoud et al., 2018). These herbs mentioned above are mainly in allusion to the pathogenic state of “stasis.”

STZ is a highly selective pancreatic islet β-cell-cytotoxic agent. There are two protocols for induction of diabetic mouse models using STZ: repeated low dose STZ and single high dose STZ (Furman, 2015). In this study, we adopted a diabetic model induced using a HFD and repeated low dose STZ, which has been reported previously (Furman, 2015; Song et al., 2018). We found that HFD led to a slight rise in mouse blood glucose. To ensure the success of the model, we adopted an STZ dose of 50 mg/kg, although 40 mg/kg is recommended in the literature (intraperitoneally, for five consecutive days). Our results show that almost all mice injected with STZ (except one) achieved an FBG level around 20 mM. Additionally, only five deaths occurred during or after model generation; however, the high blood glucose acquired slowly decreased over time in model group mice, indicating that the mice may have some ability to self-heal HFD/STZ-induced high blood glucose. After modeling, despite this possible self-healing ability, mice exhibited a series of manifestations of DN, including polydipsia, polyphagia, polyuria and body weight loss, massive proteinuria, and impaired renal function; however, those manifestations could be effectively ameliorated by BSHX decoction, particularly at a high dose. High dose BSHX decoction exerted a more stable and more significant control of blood glucose, compared with low and medium doses. This effect was similar with respect to the other manifestations of DN. Although, metformin has a similar therapeutic effect on DN, it still has a better effect in some aspects such as control of FBG and body weight loss. We speculate that a superior effect may be achieved by using a higher dose of BSHX decoction.

In addition to its therapeutic effect on target organs, Chinese medicine may also have side effects in other organ systems. A study evaluating the safety of *Carthami Flos* showed that its extract has a teratogenic effect on central nervous system development in mice, as well as a concentration-dependent cytotoxic effect (Nobakht et al., 2000). Studies on *Angelicae Pubescentis Radix* suggest that the administration of a large dose of its extract may lead to cyanosis, agitated activity, and a quickened respiration in mice and may also have nephrotoxic effects (Lu et al., 2020). Psoralen and isopsoralen are the main toxic constituents of *Psoraleae Fructus* and both elicit toxic reactions in the rat liver when administered orally (80 and 40 mg/kg, respectively) (Wang et al., 2019c). These results indicate that BSHX decoction can exert cytotoxic effects in different organ systems, especially the respiratory system, liver, and kidney; however, we noticed that these side effects were highly dose-dependent. Although the administration of higher medication doses may elicit better therapeutic effects, it also increases the risk of side effects in other organ systems. However, we adopted a normal BSHX decoction dose in this study, and the associated side effects may be tiny and has no obvious side effect report of BSHX decoction and its herbs at the normal dose. Nevertheless, because higher doses of BSHX decoction may elicit a superior therapeutic effect, identifying an optimal dose that can balance therapeutic effects and side effects merits further investigation.

Traditional Chinese herbs that perform well in experimental and clinical practice characteristically have multiple components; however, the chemical ingredients may not be clear. Using UPLC-Q-TOF-MS, we identified 67 compounds of BSHX decoction, including 20 compounds that were considered to be likely active compounds. The active compounds were as follows: hydroxysafflor yellow A, loganin, methyl linoleate, bakuchicin, eucommiol, commiferin, columbianetin, isopimpinellin, nodakenetin, psoralen, corylin, angelicin, angelol B/D/K, angelol A, angelol C/L, neobavaisoflavone, and isoneobavaisoflavone*.* In addition to providing a better understanding of the constituents of BSHX decoction, this analysis will also facilitate quality control. Furthermore, these data suggest a direction for exploring the individual components of BSHX decoction. As a main chemical component of *Carthami Flos*, Hydroxysafflor yellow A has been demonstrated to be able to reduce high glucose-induced pancreatic β-cells apoptosis via PI3K/AKT and JNK/c-Jun signaling pathways (Zhao et al., 2018; Lee et al., 2020a), and exerts a renal protective effect by inhibiting oxidative stress, reducing inflammatory responses, and attenuating renal cell apoptosis in rats with a HFD- and STZ-induced diabetes (Lee et al., 2020b). Loganin, a major chemical component of *Corni Fructus*, had the highest concentration in the medicated serum in this study, and exerts an early renal protective role in DN by inhibiting connective tissue growth factor (Jiang et al., 2012), and alleviating the loss of podocytes induced by high glucose via targeting AGEs-RAGE and its downstream pathways, p38MAPK and Nox4, *in vivo* or *in vitro* (Chen et al., 2020). Further, loganin, morroniside, and ursolic acid from *Corni Fructus* have synergistic hypoglycemic activity (He et al., 2016). Those studies reveled that there are numerous compounds in BSHX decoction that have an anti-DN role, involving multiple pathological processes and numerous targets. Nevertheless, there are no research data related to various probable active compounds of BSHX decoction, including methyl linoleate, bakuchicin, columbianetin, isopimpinellin, nodakenetin, corylin, angelol, neobavaisoflavone, isoneobavaisoflavone*,* and further experiments are needed to determine their effects or co-effects and to explore the potential roles of these novel compounds in DN.

Podocyte foot processes attach themselves to the glomerular capillaries at the glomerular basement membrane, forming intercellular junctions as slit diaphragm filtration barriers (Garg, 2018). Microproteinuria is the most prominent clinical manifestation at the early stage of DN; however, podocyte density is considered as the best predictor of proteinuria and disease progression (Meyer et al., 1999; Steffes et al., 2001), and massive proteinuria and glomerulosclerosis, progressing to tubulointerstitial fibrosis and end-stage kidney failure, is closely associated with podocyte loss or dysfunction (Matsusaka et al., 2005; Wharram et al., 2005). As podocytes are terminally differentiated cells that cannot replicate or regenerate in adults, their injury may be the weakest common link leading to glomerulosclerosis and DN (Reidy et al., 2014). The mechanisms of podocyte injury mainly include podocyte hypertrophy, EMT, podocyte detachment, and podocyte apoptosis (Dai et al., 2017). EMT was proposed as a potential explanation for podocyte depletion in DN (Yamaguchi et al., 2009). During EMT, podocytes lose their hallmark epithelial characteristics and gain features of mesenchymal cells (Ying and Wu, 2017). Our study showed that, in the DN model group, the podocyte markers, nephrin and podocin, were down-regulated, while mesenchymal markers, α-SMA and FSP-1, were up-regulated; however, the changes in these markers were reversed following treatment with BSHX decoction. Hence, we conclude that podocyte EMT occurring during DN can be effectively alleviated by treatment with BSHX decoction.

Ras-related C3 botulinum toxin substrate 1 (Rac1) is a small G protein member of the Rho family. Rac1 has a Rho type GTP binding region, which is inactivated by GDP (guanosine diphosphate) bounding and activated in a GTP (guanosine triphosphate)-bound state, which is essential for effective signaling to elicit downstream biological functions (Abdrabou and Wang, 2018). Rac1 is an important mediator of nephrin-induced signaling cascades, and may contribute to podocyte differentiation (Mouawad et al., 2013). In salt-sensitive rodent hypertension models, high-salt loading activates Rac1 in the kidneys, leading to blood pressure elevation and renal injury, presenting as severe proteinuria and extensive podocyte damage (Shibata et al., 2011). Rac1 activation switches podocytes toward a more mesenchymal phenotype, accompanied by loss of WT-1 and nephrin and induction of α-SMA and fibronectin expression (Babelova et al., 2013). In this study, GTP-Rac1 was notably overexpressed in the DN model group, and this was significantly decreased by treatment with BSHX decoction, suggesting a pharmacological effect of BSHX decoction on Rac1 activation; however, which constituent compound of BSHX decoction participates in this activity requires further investigation. p-21 activated kinase 1 (PAK1) and mitogen activated protein kinase (MAPK) are the major downstream effector kinases of Rac1 (Hou et al., 2004; Gonzalez-Villasana et al., 2015). Together with its downstream pathway, Rac1/PAK1 signaling can promote podocyte EMT under high glucose conditions, potentially via triggering β-catenin and Snail nuclear transactivation (Lv et al., 2013). p38MAPK is activated in podocytes upon induction with a constitutively active form of Rac1, while a p38 inhibitor can attenuate proteinuria, podocyte loss, and glomerulosclerosis (Robins et al., 2017). Our data show that PAK1 and p38MAPK phosphorylation are concomitant with Rac1 activation in DN model mice, and this phosphorylation was significantly reduced after treatment with BSHX decoction. The expression levels of β-catenin and Snail in the Rac1 axis were similarly changed, in that they were overexpressed in model mice and decreased in response to BSHX decoction, indicating that podocyte EMT is associated with the activation of Rac1/PAK1/p38MAPK signaling and that BSHX decoction may regulate this process via Rac1 and its downstream signaling, to alleviate renal function deficit and podocyte EMT in DN mice. As BSHX decoction may act on multiple targets, understanding the entire mechanism underlying the activity of BSHX decoction in treating DN requires considerable further experimental work to determine its effects on podocyte autophagy and apoptosis, inflammation, and oxidative stress, among other processes.

Overall, this study demonstrates that BSHX decoction can effectively ameliorate high blood glucose and renal function in DN model mice, and provides an analysis of its therapeutic substances, conducted using UPLC-Q-TOF-MS to determine the main chemical ingredients of BSHX decoction and its active compounds. Furthermore, we showed that BSHX decoction can alleviate podocyte EMT in DN mice, likely through inhibiting Rac1/PAK1/p38MAPK signaling, demonstrating its underlying mechanism. These findings regarding the active compounds and mechanism of BSHX decoction are an important step toward the exploration of the effects of the individual compounds of BSHX decoction on DN, and increase the potential for finding novel compounds in this context. Nevertheless, this study is far from sufficient; more investigations are needed and a series studies are under way in our laboratory.

## Data Availability Statement

The raw data supporting the conclusions of this article will be made available by the authors, without undue reservation.

## Ethics Statement

The animal study was reviewed and approved by the Ethics Committee for Experimental Animals of The First Hospital of Hunan University of Chinese Medicine.

## Author Contributions

WHu, DX, and WW conceived and designed the experiments. WW, WHuang, and TZ performed the experiments. HL guided and contributed to the data analysis. JL guided the animal experiment work. WW wrote the article and LX and CC assisted in this work. WHu and DX read and revised the article.

## Funding

This study was supported by the Natural Science Foundation of Hunan Province (2020JJ8024), the Open Fund of Key Laboratory of the Ministry of Education of China for Prevention and Transformation of Major Diseases in Internal Medicine of TCM (ZYNK201704), and the Scientific Research Fund Project of Hunan University of Chinese Medicine (ZYYDX201742).

## Conflict of Interest

The authors declare that the research was conducted in the absence of any commercial or financial relationships that could be construed as a potential conflict of interest.
