# Supplementary material for: Bu-Shen-Huo-Xue Decoction Ameliorates Diabetic Nephropathy by Inhibiting Rac1/PAK1/p38MAPK Signaling Pathway in High-Fat Diet/Streptozotocin-Induced Diabetic Mice
Source: Front Pharmacol. 2020 Dec 3;11:587663. doi: 10.3389/fphar.2020.587663 (PMC7744471; doi:10.3389/fphar.2020.587663)
Supplement: Supplementary file 1 [file table1.docx]

***Supplementary Material***

Supplementary table 1 Chemical compositions information of BSHX decoction

| NO. | t_R_ (min) | Aduct | Extraction mass (Da) | Error  (ppm) | Formula | Identification | Fragments | source |
| --- | --- | --- | --- | --- | --- | --- | --- | --- |
| 1 | 2.457 | + | 152.0109 | 0.71 | C_8_H_8_O_3_ | Anisic acid | (+) 153.0190, 137.0577, 121.0509, 94.0413, 66.0454 | *Angelica Sinensis Radix* (Cao, 2019) |
| 2 | 2.631 | - | 362.0432 | 0.38 | C_15_H_22_O_10_ | Catalpol | (-) 361.1135, 295.1033, 211.0722, 142.0506, 119.0364 | *Rehmanniae Radix Praeparata* (Zhang et al., 2016) |
| 3 | 3.14 | +/- | 148.0517 | 4.67 | C_5_H_8_O_5_ | Methylmalic acid | (+) 166.0868[+NH_4_^+^], 120.0747, 103.0495, 93.0638  (-) 164.0668[+NH_4_^+^], 133.6885, 115.8804 96.2900, 72.0096 | *Corni Fructus* (Zhang et al., 2009) |
| 4 | 3.865 | +/- | 293.0898 | 0.45 | C_10_H_13_N_5_O_5_ | Guanosine | (+) 311.1395[+NH_4_^+^], 232.1188, 182.0807, 136.0750  (-) 309.1097[+NH_4_^+^], 291.0995, 265.1198, 218.1033, 180.0668, 146.0822, 128.0355 | *Rehmanniae Radix Praeparata* (Zhang et al., 2016; Zhang et al., 2019b) |
| 5 | 4.544 | - | 374.0792 | -0.48 | C_16_H_22_O_10_ | Geniposidic acid | (-) 373.1139, 329.0879, 243.1235, 211.0611, 167.0348, 129.0558 | *Eucommia Cortex* (Luo et al., 2019) |
| 6 | 5.5 | +/- | 517.1553 | 0.05 | C_22_H_28_O_14_ | 3,5-Dicaffeoyl quinic acid | (+) 517.1557, 367.1493, 221.1165, 163.0379, 149.0233, 127.0381  (-)515.1419, 191.0560, 173.0455, 93.0344 | *Cortex Eucommia* (Luo et al., 2019) |
| 7 | 5.943 | + | 187.0629 | 2.19 | C_9_H_16_O_4_ | Butylidene phthalide | (+)188.0722, 163.0398, 149.0673, 127.0422, 118.0824, 109.0621 | *Angelica Sinensis Radix* (Li et al., 2019) |
| 8 | 6.042 | - | 376.0953 | 0.07 | C_20_H_24_O_7_ | Olivil | (-)375.1062, 169.0872, 151.0761, 125.0604, 101.0246, 89.0246, 59.0139 | *Eucommia Cortex* (Luo et al., 2019) |
| 9 | 6.394 | - | 164.0056 | -6.86 | C_9_H_8_O_3_ | p-Coumaric acid | (-) 163.0354, 149.0212, 119.0501, 93.0344, 83.0154 | *Lycii Fructus* (Yuan et al., 2016; Wei et al., 2018), *Cuscutae Semen* (Chen et al., 2018) |
| 10 | 6.482 | - | 516.1115 | 0.11 | C_25_H_24_O_12_ | Isochlorogenic acid | (-) 515.1031, 353.0862, 191.0578, 173.0454, 135.0448, 93.0350 | *Eucommia Cortex* (Luo et al., 2019) |
| 11 | 7.001 | - | 376.0953 | 0.16 | C_16_H_24_O_10_ | 8-Epicarboxylic acid | (-) 375.1062, 213.0764, 169.0872, 151.0769, 113.0245, 95.0504, 89.0245, 59.0139 | *Rehmanniae Radix Praeparata* (Zhang et al., 2016) |
| 12 | 7.374 | - | 516.112 | 1.62 | C_25_H_24_O_12_ | Dicaffeoylquinic acids | (-)-:515.1031, 323.0784, 191.0562, 179.0357, 161.0241 | *Lycii Fructus* (Yuan et al., 2016; Wei et al., 2018) |
| 13^*^ | 7.655 | + | 226.0735 | 3.27 | C_11_H_14_O_5_ | Genipin | (+)227.0960, 209.0652, 195.0514, 183.0494, 139.0288 | *Eucommia Cortex* (Luo et al., 2019) |
| 14 | 8.161 | +/- | 162.0314 | 1.71 | C_9_H_6_O_3_ | Umbelliferone | (+) 163.0352, 123.0806, 85.0303, 61.2268  (-) 161.0239, 133.0290, 117.0346, 105.0341, 89.0394, 77.0401, 65.0034 | *Angelicae Pubescentis Radix* (Sun et al., 2014; Wan et al., 2019) |
| 15^*▲^ | 8.512 | +/- | 612.1694 | -0.52 | C_27_H_32_O_16_ | Hydroxysafflor yellow A | (+) 613.1768, 451.1001, 433.0912, 355.0592, 313.0693, 211.0224, 181.0138, 147.0472  (-) 611.1612, 491.1193, 473.1082, 403.1031, 325.0718, 283.0608, 207.0508, 163.0036 | *Carthami Flos* (Committee, 2015; He et al., 2017) |
| 16 | 10.121 | - | 488.1157 | -0.94 | C_21_H_28_O_13_ | Cistanoside F | (-) 487.1454, 470.2291, 401.1456, 277.0352, 135.0449, 119.0365 | *Rehmanniae Radix Praeparata* (Xue et al., 2018) |
| 17^*▲^ | 10.672 | +/- | 390.1317 | 2.94 | C_17_H_26_O_10_ | Loganin | (+) 413.1381[+Na^+^], 341.1189, 229.0661, 179.0661, 161.0551, 133.0603, 109.0607  (-) 435.1514[+HCOO^-^], 227.0932, 127.0403, 101.0246, 68.9984 | *Corni Fructus* (Committee, 2015) |
| 18^*^ | 11.254 | + | 294.1102 | 0.5 | C_19_H_34_O_2_ | Methyl linoleate | (+) 295.1098, 219.0548, 206.0525, 191.0306, 175.0360, 160.0477, 132.0520, 107.0468, 72.0530 | *Lycii Fructus* (Shen et al., 2009) |
| 19^*^ | 12.158 | + | 186.0314 | 1.75 | C_11_H_6_O_3_ | Bakuchicin | (+) 187.0379, 159.0430, 143.0476, 131.0485, 115.0531, 103.0537, 77.0375 | *Psoraleae Fructus* (Wei et al., 2019) |
| 20^*^ | 12.75 | +/ | 186.0312 | 2.9 | C_9_H_12_O_4_ | Eucommiol | (+) 187.0372, 131.0496, 115.0544, 103.0545, 77.0388 | *Eucommia Cortex* (Luo et al., 2019) |
| 21 | 13.108 | + | 286.0952 | 0.71 | C_15_H_10_O_6_ | Kaempferol | (+) 287.1932, 260.0923, 217.0969, 163.0400, 121.0509 | *Eucommia Cortex* (Luo et al., 2019), *Lycii Fructus* (Yuan et al., 2016; Wei et al., 2018), *Cuscutae Semen* (Zhang et al., 2019a) |
| 22^*^ | 13.245 | + | 248.1407 | 2.09 | C_15_H_20_O_3_ | Commiferin | (+) 249.1457, 135.0772, 123.0783, 105.0677, 91.0544, 67.0531, 55.0541 | *Myrrh* (Zhao et al., 2011) |
| 23 | 13.322 | - | 382.1578 | 2.38 | C_25_H_50_O_2_ | Methyl Tetracosanoate | (-) 381.1909, 337.2023, 245.1892, 135.0402, 101.0597, 88.0486, 69.0350 | *Lycii Fructus* (Shen et al., 2009) |
| 24 | 14.301 | + | 302.0425 | 0.43 | C_15_H_10_O_7_ | Quercetin | (+) 303.0502, 281.1817, 265.1418, 249.1403,235.1673, 221.1160, 189.0544 | *Lycii Fructus* (Yuan et al., 2016; Wei et al., 2018), *Cuscutae Semen* (Zhang et al., 2019a), *Corni Fructus* (Zhang et al., 2009; Liu et al., 2011), *Carthami Flos* (He et al., 2017) |
| 25 | 14.72 | - | 610.1226 | -0.46 | C_27_H_30_O_16_ | Rutin | (-) 609.1458, 553.1592, 465.1169, 404.1648, 300.0296, 271.0179, 216.8916, 138.0615, 94.0436 | *Carthami Flos* (He et al., 2017) |
| 26^▲^ | 15.063 | +/- | 464.0956 | -0.27 | C_21_H_20_O_12_ | Hyperoside | (+) 487.2090[+Na^+^], 445.2313, 381.1507, 355.1578, 292.1495,258.1369, 211.0456, 191.1086,163.0368, 127.0381, 91.0335  (-) 463.0881, 367.1039, 345.1557, 205.0509, 161.0609, 112.9856 | *Cuscutae Semen* (Committee, 2015) |
| 27 | 15.256 | + | 302.0424 | 0.86 | C_15_H_10_O_7_ | 6-Hydroxykaempferol | (+) 303.0498, 267.1588, 249.1480, 221.1166, 181.0510, 163.0388, 149.0227, 121.0509 | *Carthami Flos* (Wang et al., 2015) |
| 28^*^ | 15.481 | + | 246.0889 | 1.43 | C_14_H_14_O_4_ | Columbianetin | (+) 247.0874, 229.0757, 214.0496, 175.0305, 135.0666, 123.0752, 103.0468 | *Angelicae Radix Pubescentis* (Wan et al., 2019) |
| 29 | 15.886 | +/- | 594.1588 | -0.49 | C_27_H_30_O_15_ | Kaempferol-3-rutinoside | (+) 595.1641, 538.2280, 341.1384, 293.1727, 265.0977, 249.1485, 221.1176, 167.1062, 121.0509  (-) 593.1512, 569.1873, 519.1873, 427.1970, 357.1344, 151.0402 | *Lycii Fructus* (Peng and Tian, 2001) |
| 30 | 16.007 | + | 624.1692 | -0.2 | C_28_H_32_O_16_ | Verbascoside/Isoverbascoside | (+) 625.3029, 583.5243, 479.1191, 317.0679, 129.0602, 85.0380 | *Rehmanniae Radix Praeparata* (Committee, 2015; Xue et al., 2018), *Cistanches Herba* (Committee, 2015; Gao et al., 2019) |
| 31^*^ | 16.133 | + | 246.089 | 0.95 | C_13_H_10_O_5_ | Isopimpinellin | (+) 247.0932, 175.0373, 147.0421, 131.0474, 119.0473, 91.0530, 77.0371, 65.0370 | *Angelicae Radix Pubescentis* (Wan et al., 2019) |
| 32 | 16.452 | +/- | 448.1003 | 0.51 | C_21_H_20_O_11_ | Quercitrin/ Isoquercitrin | (+) 449.1082, 373.1747, 345.1304, 3275.1600, 252.1969, 235.1668, 175.1110, 143.1056, 121.0509  (-) 447.0893, 284.0327, 255.0300, 227.0349 | *Lycii Fructus* (Peng and Tian, 2001; Wei et al., 2018), *Cuscutae Semen* (Zhang et al., 2019a), *Corni Fructus* (Zhang et al., 2009) |
| 33 | 18.169 | + | 278.1151 | 0.99 | C_15_H_18_O_5_ | Ulopterol | (+) 279.1486, 261.1523, 229.1527, 189.0914, 161.0778, 115.0710, 71.0384 | *Angelicae Radix Pubescentis* (Wan et al., 2019) |
| 34 | 18.884 | +/- | 354.1102 | 0.45 | C_16_H_18_O_9_ | Chlorogenic Acid | (+) 355.1175, 279.0644, 221.1164, 147.0438, 121.0509  (-) 353.1040, 335.0916, 295.0617, 234.4548, 161.0238, 108.9891, 59.4035 | *Eucommia Cortex* (Luo et al., 2019), *Lycii Fructus* (Yuan et al., 2016; Wei et al., 2018), *Cuscutae Semen* (Qu et al., 2014), *Angelica Radix Sinensis* (Li et al., 2019) |
| 35^*^ | 19.84 | + | 246.089 | 1 | C_14_H_14_O_4_ | Nodakenetin | (+) 247.0932, 175.0384, 159.0429, 147.0431, 131.0480, 119.0478, 91.0534 | *Angelicae Pubescentis Radix* (Wan et al., 2019) |
| 36 | 21.913 | - | 328.1823 | 1.17 | C_18_H_16_O_6_ | Corylisoflavone A | (-) 327.2178, 259.0978, 209.1182, 160.8423, 112.9856 | *Psoraleae Fructus* (Wei et al., 2019) |
| 37^*▲^ | 22.582 | + | 186.0313 | 2.27 | C_11_H_6_O_3_ | Psoralen | (+) 187.0299, 159.0352, 143.0420, 131.0422, 115.0478, 77.0338 | *Psoraleae Fructus* (Wei et al., 2019) |
| 38^*^ | 22.869 | + | 320.1033 | 4.76 | C_20_H_16_O_4_ | Corylin | (+) 321.0983, 303.0915, 279.0545, 211.0673, 183.0742, 163.0305, 137.0177 | *Psoraleae Fructus* (Wei et al., 2019) |
| 39^*▲^ | 23.106 | + | 186.0313 | 2.24 | C_11_H_6_O_3_ | Angelicin | (+) 187.0372, 131.0500, 115.0545, 103.0546, 77.0384 | *Psoraleae Fructus* (Wei et al., 2019) |
| 40^*^ | 23.293 | + | 376.1521 | 0.16 | C_20_H_24_O_7_ | Angelol B /D/ K | (+) 377.1567, 259.0933, 219.0600, 205.0462 | *Angelicae Pubescentis Radix* (Wan et al., 2019) |
| 41^*▲^ | 23.594 | + | 376.1521 | 0.3 | C_20_H_24_O_7_ | Angelol A | (+) 377.1481, 219.0663, 205.0507, 191.0353, 175.0391, 160.0526 | *Angelicae Pubescentis Radix*(Wan et al., 2019) |
| 42^*^ | 23.756 | + | 378.1676 | 0.57 | C_20_H_26_O_7_ | Angelol C/L | (+) 379.1684, 259.0916, 219.0610, 205.0453, 191.0300, 160.0483, 147.0409, 131.0465, 57.0686 | *Angelicae Pubescentis Radix* (Wan et al., 2019) |
| 43^*^ | 23.947 | + | 378.1677 | 0.36 | C_20_H_26_O_7_ | Angelol C/L | (+) 379.1652, 277.0981, 206.0517, 160.0478, 85.0627, 57.0689 | *Angelicae Pubescentis Radix* (Wan et al., 2019) |
| 44^▲^ | 24.16 | + | 376.1518 | 0.99 | C_20_H_24_O_7_ | Angelol G | (+) 377.1631, 259.0986, 231.1034, 219.0670, 205.0510, 83.0492, 55.0542 | *Angelicae Pubescentis Radix* (Wan et al., 2019) |
| 45 | 24.457 | + | 376.1677 | 0.4 | C_20_H_26_O_7_ | Angelol B /D/ K | (+) 377.1616, 259.0877, 231.0926, 160.0458, 57.0680 | *Angelicae Pubescentis Radix* (Wan et al., 2019) |
| 46^*^ | 24.628 | + | 378.1675 | 0.84 | C_20_H_24_O_7_ | Angelol C/L | (+) 379.1765, 259.0957, 227.0690, 206.0560, 191.0314, 175.0361 | *Angelicae Pubescentis Radix* (Wan et al., 2019) |
| 47 | 27.352 | +/- | 324.1364 | -0.64 | C_20_H_20_O_4_ | Bavachin / Isobavachin | (+) 325.1413, 269.0780, 149.0216  (-) 323.1100, 203.0717, 159.0816, 119.0503 | *Psoraleae Fructus* (Wei et al., 2019) |
| 48 | 27.685 | +/- | 340.131 | 0.1 | C_20_H_20_O_5_ | Isobavachin | (+) 341.1374, 323.1282, 305.1190, 269.0801, 251.0691, 221.0691, 203.0683, 149.0226  (-) 339.1241, 287.1651, 119.0500 | *Psoraleae Fructus* (Wei et al., 2019) |
| 49 | 27.831 | + | 228.0785 | 0.71 | C_14_H_12_O_3_ | Angenomalin/Isoangenomalin | (+) 229.0853, 214.0587, 187.0386, 175.0384, 159.0435, 131.0483 | *Angelicae Pubescentis Radix* (Wan et al., 2019) |
| 50^*▲^ | 28.388 | +/- | 322.1203 | 0.57 | C_20_H_18_O_4_ | Neobavaisoflavone | (+) 323.1236, 255.0698, 199.0789, 165.0727, 137.0258, 103.0562, 69.0713  (-) 321.1139, 277.0509, 265.0515, 237.0562, 223.0418 | *Psoraleae Fructus* (Wei et al., 2019) |
| 51^*^ | 28.545 | +/- | 322.1206 | -0.21 | C_20_H_18_O_4_ | Isoneobavaisoflavone | (+) 323.1236, 267.0725, 239.0763, 195.0487, 107.0513, 69.0708  (-) 321.1128, 266.0584, 187.1133, 132.0583, 91.0195 | *Psoraleae Fructus* (Wei et al., 2019) |
| 52 | 28.751 | - | 356.1078 | 1.3 | C_20_H_20_O_6_ | Brosimacutin G | (-) 355.0966, 327.1233, 283.1362, 193.0870, 151.0768, 124.0163, 57.0348 | *Psoraleae Fructus* (Ding et al., 2018) |
| 53 | 28.962 | - | 288.1538 | 3.49 | C_16_H_16_O_5_ | Angelica Ketone | (+) 287.1652, 271.1338, 241.1258, 213.0898, 175.0746, 77.0390 | *Angelica Sinensis Radix* (Cao, 2019) |
| 54 | 29.374 | +/- | 324.1362 | 0 | C_20_H_20_O_4_ | Bavachin / Isobavachin | (+) 325.1361, 269.0878, 205.0889, 149.0271, 121.0342, 93.0365, 69.0716  (-) 323.1100, 203.0710, 159.0805, 119.0504 | *Psoraleae Fructus* (Wei et al., 2019) |
| 55 | 29.89 | + | 220.1825 | 1.04 | C_15_H_24_O | Caryophyllene oxide | (+) 221.1905, 203.1178, 147.1165, 135.1147, 107.0836, 71.0478, 55.0519 | *Angelica Sinensis Radix* (Li et al., 2019), *Myrrh* (Zhao et al., 2011) |
| 56 | 30.083 | + | 172.0884 | 2.26 | C_9_H_16_O_3_ | 1‑Deoxyeucommiol | (+) 173.0958, 157.0679, 144.0589, 128.0641, 115.0561, 77.0401 | *Eucommia Cortex* (Luo et al., 2019) |
| 57 | 31.61 | +/- | 324.1362 | -0.22 | C_20_H_20_O_4_ | Bavachalcone | (+) 325.1469, 269.0820, 227.0694, 205.0884, 149.0247, 121.0317  (-) 323.1100, 203.0704, 159.0816, 119.0505 | *Psoraleae Fructus* (Wei et al., 2019) |
| 58 | 31.758 | +/- | 336.0997 | 0.21 | C_20_H_16_O_5_ | Psoralidin/Isopsoralidin | (+) 337.0901, 309.1004, 281.0303, 253.0435, 209.0838, 150.1238  (-) 335.0931, 319.0634, 280.0375 | *Psoraleae Fructus* (Wei et al., 2019) |
| 59 | 32.561 | + | 190.0987 | 3.51 | C_12_H_14_O_2_ | Z-Ligustilide | (+) 191.1048, 128.0579, 115.0517, 91.0528, 77.0368, 65.0374 | *Angelica Sinensis Radix* (Li et al., 2019) |
| 60^▲^ | 33.014 | + | 244.1099 | 0.13 | C_15_H_16_O_3_ | Osthole | (+) 245.1177, 189.0558, 131.0494, 103.05537, 77.0384 | *Angelicae Pubescentis Radix* (Sun et al., 2014; Committee, 2015) |
| 61 | 33.622 | +/- | 324.1361 | 0.09 | C_20_H_20_O_4_ | Corylifolinin | (+) 325.1442, 269.0814, 149.0235, 121.0509, 93.0298, 65.0371  (-) 323.1100, 203.0717, 159.0817, 119.0508 | *Psoraleae Fructus* (Wei et al., 2019) |
| 62 | 34.336 | + | 328.131 | 0.1 | C_19_H_20_O_5_ | Columbianadin | (+) 329.1243, 229.0927, 187.0439, 175.0439, 159.0480, 101.0620 | *Angelicae Pubescentis Radix* (Committee, 2015; Wan et al., 2019) |
| 63 | 34.512 | +/- | 338.1521 | -0.99 | C_20_H_18_O_5_ | Psoralenol | (+) 339.1499, 271.1167, 219.1177, 177.0664, 147.0550, 119.0576, 91.0603, 69.0749  (-) 337.1244, 163.1135, 119.0501 | *Psoraleae Fructus* (Wei et al., 2019) |
| 64 | 35.084 | +/- | 390.183 | 0.3 | C_17_H_26_O_10_ | Corylifol A | (+) 391.1969, 267.0875, 239.0918, 211.0934, 183.0931, 137.0339, 69.0744  (-) 389.1624, 320.0920, 303.0977, 291.0599, 277.0414, 265.0417 | *Psoraleae Fructus* (Wei et al., 2019) |
| 65 | 37.043 | - | 272.1587 | 3.52 | C_15_H_12_O_5_ | Naringenin | (-) 271.1566, 203.0097, 188.0824, 172.0518, 135.0450, 108.0218 | *Psoraleae Fructus* (Zhang et al., 2009) |
| 66 | 37.37 | +/- | 338.1518 | 0.03 | C_16_H_18_O_8_ | 3-p-Coumaroyl-quinic acid | (+) 339.1499, 219.1036, 177.0579, 161.0578, 147.0452, 119.0511, 91.0545, 69.0705  (-) 337.1244, 119.0508, 65.0396 | *Eucommia Cortex* (Luo et al., 2019) |
| 67 | 39.739 | - | 256.164 | 3.14 | C_15_H_12_O_4_ | Liquiritigenin | (-) 255.1625, 172.0887, 156.0578, 143.0497, 119.0495, 93.0342 | *Eucommia Cortex* (Huang et al., 2019) |

Note: ^*^ Expressed as compositions which were found in the serum, ^▲^ expressed as compositions which were authenticated by standard substances.

**Reference**

Cao, Y.D. (2019). Analysis of chemical components and pharmacological action of Angelica. *World Latest Medicine Information* 19(02)**,** 93+95.

Chen, L., Zhao, L.H., and Ying, T.X. (2018). Simultaneous Determination of Flavonoid Active Components and Phenolic Acids in Semen cuscutae by HPLC *Pharmaceutical and Clinical Research* 26(02)**,** 101-103.

Committee, S.P. (2015). Pharmacopoeia of the People's Republic of China. 151.

Ding, Y.T., Zheng, Z.H., Zhao, R.Y., Zhang, N., Sun, Y.J., Li, J.H., et al. (2018). Rapid Identification of Chemical Constituents in Psoralea coryifolia by UPLC-Q-TOF-MS Combined with UNIFI Informatics Platform. *Journal of Chinese Mass Spectrometry Society* 39(06)**,** 729-745.

Gao, Y., Guo, L.N., Ma, S.C., Liu, J., Zheng, J., and Zan, K. (2019). Comparative studies of three Cistanche speices based on UPLC specific chromatogram and determination of main components. *China Journal of Chinese Materia Medica* 44(17)**,** 3749-3757.

He, T., Li, K.A., Ji, Z.H., and Tian, S.G. (2017). Simultaneous Determination of Four Chemical Components in Carthamus tinctorius L. by HPLC. *Chinese Journal of Information on TCM* 24(04)**,** 79-82.

Huang, W.X., Yao, T., Ding, L.Q., and Li, W. (2019). Chemical constituents from barks of Eucommia ulmoides. *Chinese Traditional and Herbal Drugs* 50(14)**,** 3279-3283.

Li, X., Li, C.Y., Qiang, Z.Z., He, J.G., Zhu, J.H., Li, B., et al. (2019). Determination of Index Components in Angelica Sinensis Radix by Diffbrent Drying Methods. *Modern Chinese Medicine* 21(08)**,** 1110-1113.

Liu, Y.K., Yu, W.W., and Bai, Y. (2011). Application of UPLC-ESI-Q-TOF-MS on Chemical Constitutents of Cornus officinalis. *Chinese Journal of Modern Applied Pharmacy* 28(03)**,** 226-230.

Luo, X.M., Su, M.F., Chang, X.Y., Wang, X.M., Li, Z.Z., Wang, W.H., et al. (2019). Qualitative and quantitative analysis of main chemical constituents in Eucommia ulmoides by LC-MS. *Modern Chinese Medicine* 21(08)**,** 1029-1040.

Peng, X.M., and Tian, G.Y. (2001). Structural characterization of the glycan part of glycoconjugate LbGp2 from Lycium barbarum L. *Carbohydrate Research* 331(1)**,** 95-99.

Qu, Y., Li, Q., Wei, W.L., and Zeng, R. (2014). HPLC-UV specific chromatogram study and determination of seven organic acids from Datusizi (Cuscutae Japonicae Semen) before and after salt processing. *Chin J Pharm Anal* 34(05)**,** 824-829.

Shen, H.L., Xiang, N.J., Gao, Q., Ni, C.M., and Miao, M.M. (2009). Analysis of Fatty Acids Components of Medlar by GC/MS. *Journal of Chinese Mass Spectrometry Society* 30(02)**,** 99-104.

Sun, D.D., Xu, X.F., Yan, S.H., Song, X.M., and Li, X. (2014). Analysis on Chemical Components from Water Extract of Angelicae pubescentis Radix by High Performance Liquid Chromatography-electrospray Ionization-quadrupole-time of Flight-mass Spectrometry. *Nat Prod Res Dev* 26(01)**,** 69-76.

Wan, M.Q., Zhang, Y.B., Yang, Y.F., Liu, X.Y., Jia, L.Y., and Yang, X.W. (2019). Analysis of the chemical composition of Angelicae Pubescentis Radix by ultra-performance liquid chromatography and quadrupole time-of-flight tandem mass spectrometry *Journal of Chinese Pharmaceutical Sciences* 28(03)**,** 145-159.

Wang, S.S., Ma, Y., Zhang, Y., Li, D.F., Yang, H.J., and Liang, R.X. (2015). Rapid identification of chemical composition in safflower with UHPLC-LTQ-Orbitrap. *China Journal of Chinese Materia Medica* 40(07)**,** 1347-1354.

Wei, M.M., Wang, S.Y., Yang, W., Li, Y.F., and Li, C. (2019). Chemical Constituents of Psoraleae Fructus and Its Main Toxic Ingredients. *Chinese Journal of Experimental Traditional Medical Formulae* 25(07)**,** 207-219.

Wei, X.S., Wang, H.Y., Sun, Z.Y., Sun, X.H., and Zhou, H.J. (2018). Research progress in chemical constituents and pharmacological activities of Lycii Fructus in Ningxia. *Chinese Traditional Patent Medicine* 40(11)**,** 2513-2520.

Xue, G.Q., Jin, M.L., Li, S.N., Gao, S.L., Cao, B.S., and Pan, X.Y. (2018). Chemical constituents from Rehmannia Radix Praeparata and their biological activities in vitro. *Chinese Traditional Patent Medicine* 40(12)**,** 2689-2692.

Yuan, Y.B., Wang, Y.B., Jiang, Y.M., Prasad, K.N., Yang, J.L., Qu, H.X., et al. (2016). Structure identification of a polysaccharide purified from Lycium barbarium fruit. *International Journal of Biological Macromolecules* 82**,** 696-701.

Zhang, B.Y., Jiang, Z.Z., Wang, Y.F., Yang, L., Yang, F., and Yu, H.J. (2016). Analysis of chemical constituents in fresh, dried and prepared Rehmanniae Radix by UPLC/ESI-Q-TOF MS. *Chinese Traditional Patent Medicine* 38(05)**,** 1104-1108.

Zhang, Y.E., Liu, E.H., Li, H.J., and Li, P. (2009). Chemical Constituents from the Fruit of Cornus officinalis *Chinese Journal of Natural Medicines* 7(05)**,** 365-367.

Zhang, Y.L., Meng, F.J., Tian, Y., Hou, J.P., and Zhang, X.J. (2019a). Simultaneous determination of six main active components in semen Cuscuta from different habitats by RP-HPLC. *Chemical Engineer* 33(05)**,** 29-33.

Zhang, Y.L., Yang, Y.Y., Bai, Z.Y., and Cao, Y.G. (2019b). Simultaneous determination of eight nucleosides in Rehmanniae Radix Praeparata by UPLC-MS/MS. *Chinese Journal of Pharmaceutical Analysis* 39(04)**,** 608-614.

Zhao, J.F., Zhou, C.L., Han, L., Wei, T.M., and Zhou, F.Q. (2011). Research Progress of Myrrh. *China Pharmacy* 22(07)**,** 661-665.
